# Supplementary material for: Transfer RNA-mediated restoration of potassium current and electrical correction in premature termination long-QT syndrome hERG mutants
Source: Mol Ther Nucleic Acids. 2023 Sep 16;34:102032. doi: 10.1016/j.omtn.2023.102032 (PMC10568093; doi:10.1016/j.omtn.2023.102032)
Supplement: Document S1. Figures S1–S4 and Tables S1–S7 [file mmc1.pdf]

OMTN, Volume *34*

Supplemental information

Transfer RNA-mediated restoration of potassium current and electrical correction in premature termination long-QT syndrome hERG mutants

Viggo G. Blomquist, Jacqueline Niu, Papiya Choudhury, Ahmad Al Saneh, Henry M. Colecraft, and Christopher A. Ahern

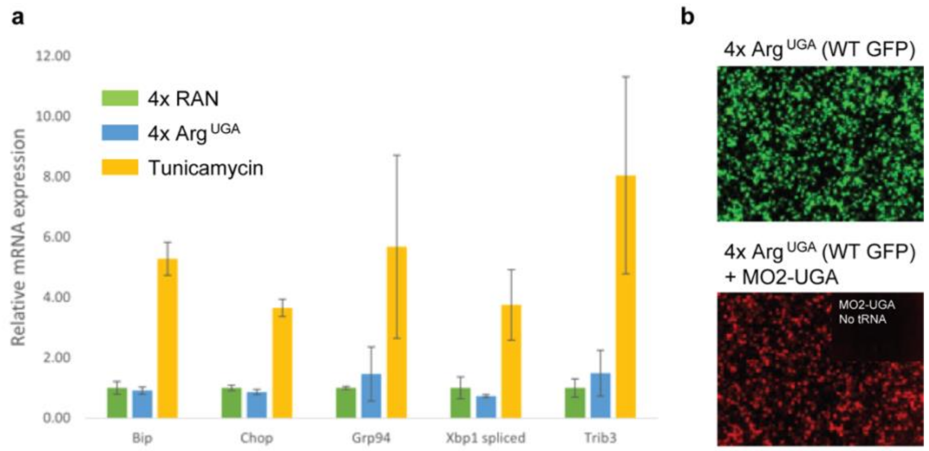

Figure S1. Expressed tRNA do not initiate UPR. a) HEK cells expressing the indicated construct were assayed by RT-PCR for UPR. 4X Arg^{UGA} contains four copies of an Arg^{UGA} suppressor, where 4X RAN has a randomized Arg^{UGA} tRNA cassette. Both plasmids contain a WT GFP to mark plasmid delivery. Tunicamycin is used as the positive control. b) upper, Expression of the 4X Arg^{UGA} GFP construct; lower, Arg^{UGA} tRNA activity as shown by rescue of an MO2-UGA plasmid.

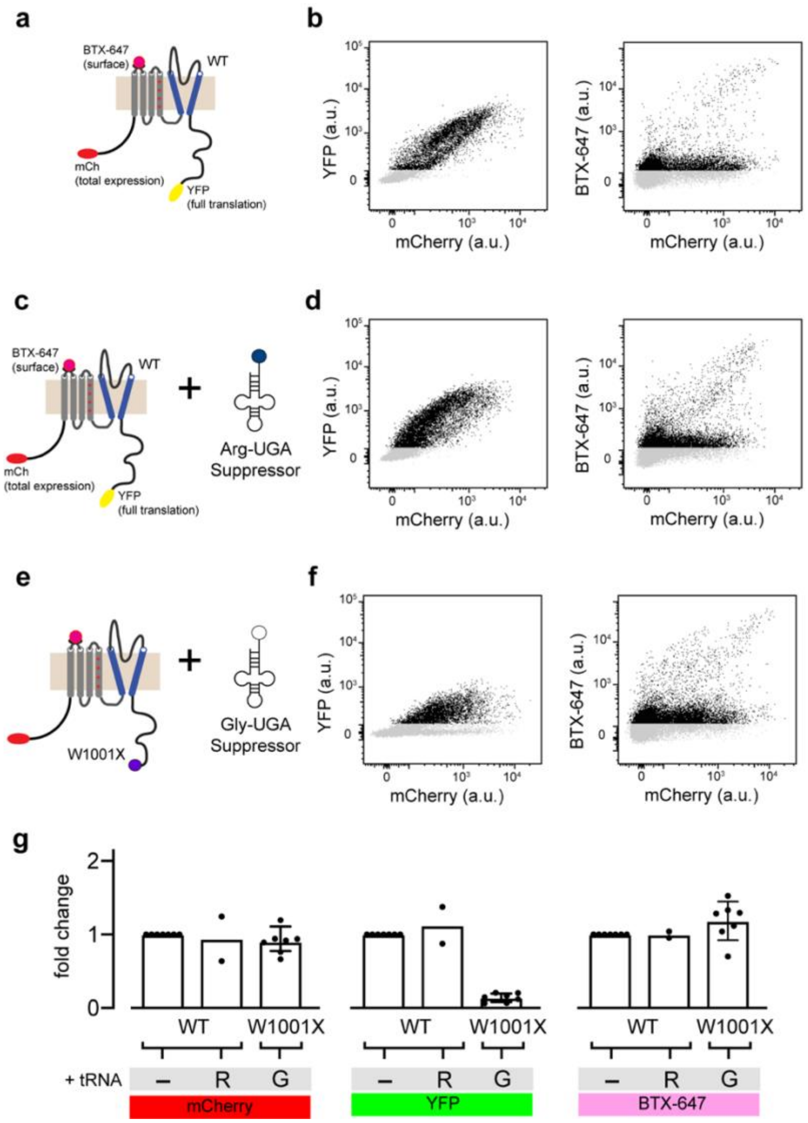

Figure S2. tRNA expression does not impact Wt hERG expression. a) Illustration of engineered WT hERG with mCherry fluorescence tag on the N-terminus, YFP on the C-terminus, and BTX-647 tagging the extracellular BTX binding site. b) Flow cytometric analysis of population distribution for mCherry, YFP, and BTX-647 fluorescence, where each cell is represented by a single point in a trial. YFP is plotted as a function of mCherry to show the relative proportion of channels fully translated (left). BTX-647 is plotted as a function of mCherry to show relative fraction of hERG trafficked to the plasma membrane (right). c) Cartoon depiction of hERG co-expressed Arg^{UGA} tRNA. d) Flow cytometric analysis of population distribution shows co-expression with tRNA does not affect mCherry (left) or channel trafficking (right). e) Cartoon depiction of hERG co-expressed Gly^{UGA} tRNA. f) Flow cytometric analysis of population distribution shows expression of tRNA restores full length hERG and YFP fluorescence (left) and has no effect on trafficking (right). g) Population summary of the geometric means of mCherry, YFP, and BTX-647 fluorescence for Wt hERG and W1001X co-expressed with tRNAs for each trial (where each trial includes >5000 cells). Error bars represent standard deviation (mean ± sd).

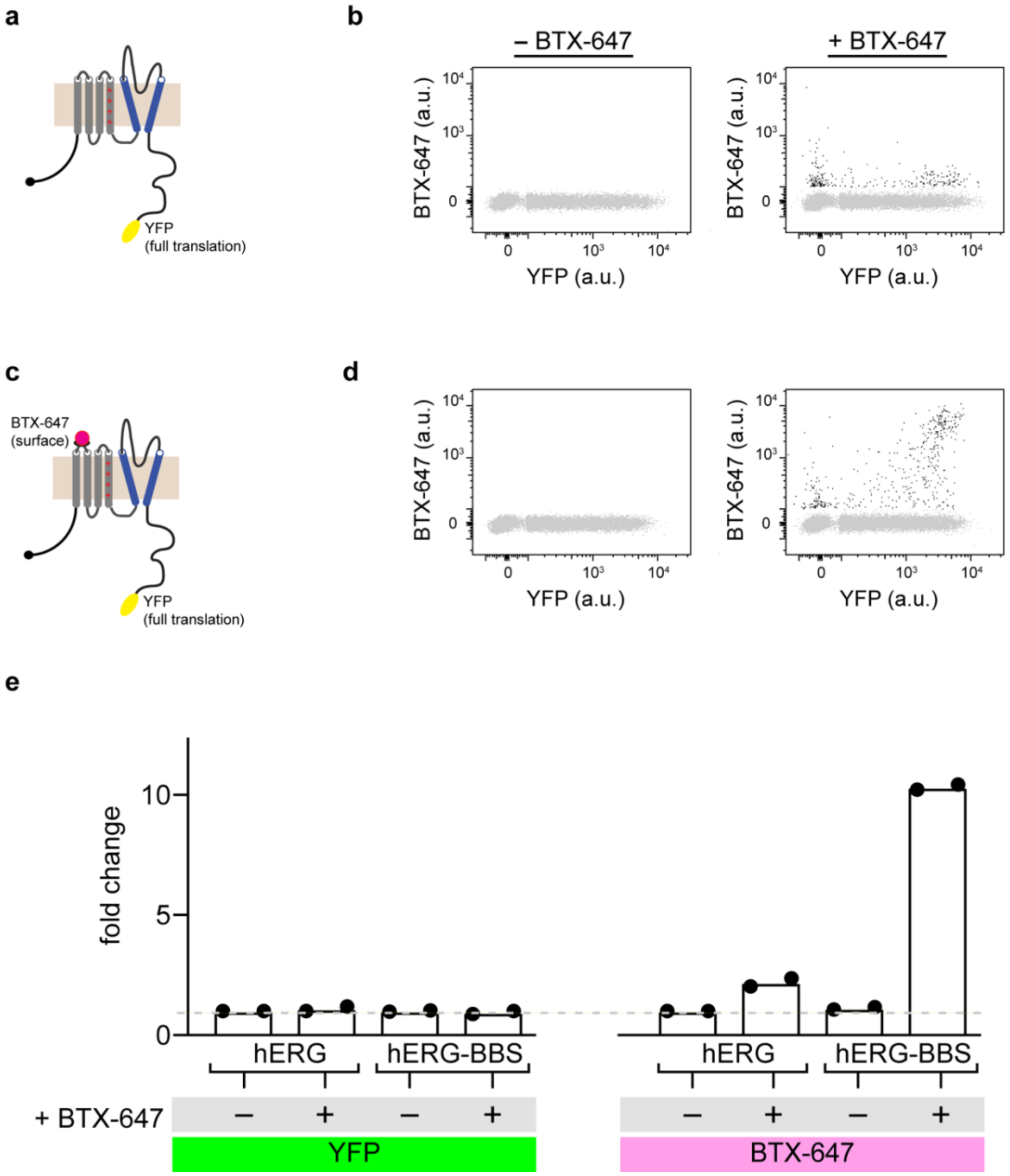

Figure S3. BTX-647 labeling is specific to BBS site in hERG. a) Illustration of engineered WT hERG with YFP fluorescence tag on the C-terminus. b) BTX-647 is plotted as a function of YFP to demonstrate any nonspecific signal without (left) or with (right) BTX-647 treatment. c) Illustration of engineered WT hERG with YFP fluorescence tag on the C-terminus and BTX-647 tagging the extracellular BTX binding site. d) BTX-647 is plotted as a function of YFP to demonstrate absence of membrane labeling without BTX-647 treatment (left) and clear labeling with BTX-647 treatment (right).

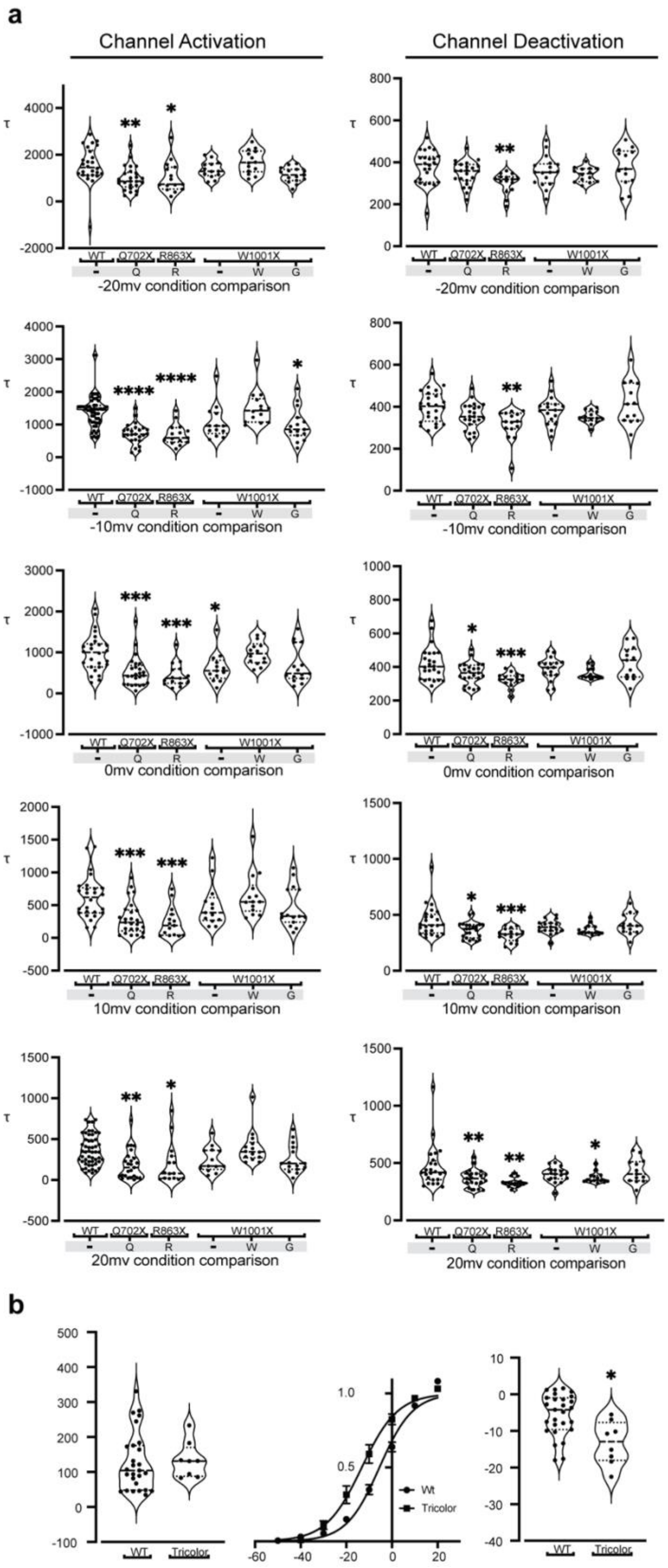

Figure S4. Further analysis of channel kinetics. a) hERG activation (left) and deactivation (right) at step voltages from -20mV to 20mV. A Dunnett's multiple comparison test was used to determine statistical significance (p>.05). b) WT and tricolor hERG current densities (left) current-voltage relationship (middle) and $V_{.05}$ values (right). An unpaired t-test was used to determine statistical significance (p>.05).

Table S1. GFP Rescue. Values are presented as mean ± SEM. The data in this table was used to generate Figure 1.

Condition (n)	GFP (normalized to Wt)
Wt GFP (3)	1.0 ± 0.0
GFP-TAG (3)	0.001 ± 0.0006
GFP-TAG + Gln tRNA (3)	0.3 ± 0.09
GFP-TGA (3)	0.002 ± 0.0005
GFP-TGA + Trp tRNA (3)	0.02 ± 0.005
GFP-TGA + Arg tRNA (3)	0.2 ± 0.05

Table S2. hERG expression, rescue, and trafficking. Values are presented as mean ± SEM. Statistical significance was set at p<0.05 and marked with asterisks. The data in this table was used to generate Figure 2 And Supplemental Figure S2.

Condition (n)	mCherry (normalized to WT)	YFP (normalized to WT)	BTX-647 (normalized to WT)
Wt hERG (11)	1.0 ± 0.0	1.0 ± 0.0	1.0 ± 0.0
Q702x (7)	2.0 ± 0.3*	0.0 ± 0.006	2.3 ± 0.2
Q702x + Gln tRNA (7)	1.0 ± 0.1	0.7 ± 0.07*	1.0 ± 0.08
R863X (7)	1.1 ± 0.1	0.0 ± 0.008	0.8 ± 0.06
R863X + Arg tRNA (7)	1.2 ± 0.1	0.6 ± 0.07*	1.1 ± 0.1
W1001X (9)	0.9 ± 0.07	0.009 ± 0.004	1.1 ± 0.1
W1001X + Trp tRNA (9)	0.9 ± 0.07	0.05 ± 0.008	1.2 ± 0.1
W1001X + Gly tRNA (9)	0.9 ± 0.05	0.1 ± 0.01	1.1 ± 0.08
Wt hERG + Arg tRNA (3)	0.9 ± 0.1	1.1 ± 0.1	1.1 ± 0.1

Table S3. Activation of rescued hERG. Values are presented as mean ± SEM. Statistical significance was set at p<0.05 and marked with asterisks.

Condition (n)	-20mV	-10mV	0mV	10mV	20mV
	τ activation (ms)	τ activation (ms)	τ activation (ms)	τ activation (ms)	τ activation (ms)
Wt hERG (26)	1549 ± 155.0	1408 ± 101.7	988.3 ± 91.4	620.4 ± 63.4	360.2 ± 36.6
Q702x + Gln tRNA (25)	976.0 ± 102.2*	721.0 ± 62.4*	521.6 ± 74.0*	300.2 ± 50.1*	187.8 ± 35.7*
R863X + Arg tRNA (15)	1032 ± 170.8*	697.5 ± 82.25*	446.5 ± 73.6*	237.3 ± 58.7*	194.0 ± 65.0*
W1001X (13)	1367 ± 100.4	1070 ± 145.6	619.8 ± 99.2*	490.6 ± 88.8	247.5 ± 42.9
W1001X + Trp tRNA (14)	1699 ± 132.9	1512 ± 139.2	989.4 ± 77.4	647.9 ± 88.7	399.0 ± 54.9
W1001X + Gly tRNA (15)	1134 ± 81.8	982.5 ± 128.7*	679.6 ± 115.6	461.6 ± 80.4	253.5 ± 45.2

Table S4. Deactivation of rescued hERG. Values are presented as mean ± SEM. Statistical significance was set at $p<0.05$ and marked with asterisks. The data in this table was used to generate Supplemental Figure S4.

Condition (n)	-20mV	-10mV	0mV	10mV	20mV
	τ deactivation (ms)	τ deactivation (ms)	τ deactivation (ms)	τ deactivation (ms)	τ deactivation (ms)
Wt hERG (24)	375.9 ± 16.3	400.9 ± 14.9	418.9 ± 20.3	442.9 ± 27.8	466.8 ± 37.9
Q702x + Gln tRNA (23)	347.6 ± 11.8	354.3 ± 12.9	362.2 ± 13.3	362.5 ± 14.9	367.0 ± 15.5
R863X + Arg tRNA (15)	302.7 ± 12.4	314.6 ± 18.3	324.2 ± 10.9	322.0 ± 15.6	330.3 ± 10.0
W1001X (17)	357.6 ± 17.3	381.3 ± 16.0	389.6 ± 16.2	390.3 ± 15.0	398.3 ± 16.2
W1001X + Trp tRNA (14)	342.0 ± 9.1	350.4 ± 7.9	363.3 ± 9.5	366.4 ± 12.2	371.9 ± 12.4
W1001X + Gly tRNA (13)	378.2 ± 25.1	419.0 ± 28.3	425.2 ± 26.8	417.3 ± 26.6	430.8 ± 29.3

Table S5. Action Potential Parameters I. Values are presented as mean ± SEM. The data in this table was used to generate Figure 4.

Condition	Resting Membrane Potential (mV)	Upstroke Velocity (V/s)	Action Potential Amplitude (mV)
blank	-69.0±4.0	88.8±72.1	112.1±27.9
WT	-61.9±2.2	85.1±40.2	103.5±18.4
R863X	-65.9±2.5	91.6±56.6	128.9±24.8
R863X+ArgtRNA	-56.5±1.8	72.9±38.4	98.1±14.0

Table S6. Action Potential Parameters: APD80 1 hz. Values are presented as mean ± SEM. Myocytes were paced at 1hz. One-way ANOVA ($P = 0.0736$) followed by Dunnett's multiple comparisons test was used to test statistical significance.

	Avg	SEM	n	Dunnett's Multiple Comparisons Test (p-values)
blank	437.11	72.41	6	0.5268
WT	339.71	37.97	7	n/a
R863X	622.50	134.5	3	0.0332
R863X+ArgtRNA	380.57	43.46	5	0.9399

Table S7. Tricolor hERG comparison to Wt hERG. Values are presented as mean ± SEM. Statistical significance was set at $p<0.05$ and marked with asterisks. The data in this table was used to generate Supplementary Figure S3.

Condition (n)	IV	Current Density
	$V_{1/2}$ (mV)	pA/pF
Wt hERG (27)	-5.5 ± 1.0	129.4 ± 16.1
Tricolor hERG (8)	-13.2 ± 2.0*	134.5 ± 18.2
