# Supplementary material for: Transfer RNA-mediated restoration of potassium current and electrical correction in premature termination long-QT syndrome hERG mutants
Source: Mol Ther Nucleic Acids. 2023 Sep 16;34:102032. doi: 10.1016/j.omtn.2023.102032 (PMC10568093; doi:10.1016/j.omtn.2023.102032)
Supplement: Document S2. Article plus supplemental information [file mmc2.pdf]

Molecular Therapy
Nucleic Acids
Original Article

Transfer RNA-mediated restoration of potassium current and electrical correction in premature termination long-QT syndrome hERG mutants

Viggo G. Blomquist,[1,3] Jacqueline Niu,[1,3] Papiya Choudhury,[1] Ahmad Al Saneh,[2] Henry M. Colecraft,[1] and Christopher A. Ahern[2]

[1]Department of Physiology and Cellular Biophysics, Columbia University Medical Center, New York, NY 10032, USA; [2]Department of Molecular Physiology and Biophysics, University of Iowa Carver College of Medicine, Iowa City, IA, USA

Disease-causing premature termination codons (PTCs) individually disrupt the functional expression of hundreds of genes and represent a pernicious clinical challenge. In the heart, loss-of-function mutations in the hERG potassium channel account for approximately 30% of long-QT syndrome arrhythmia, a lethal cardiac disorder with limited treatment options. Premature termination of ribosomal translation produces a truncated and, for potassium channels, a potentially dominant-negative protein that impairs the functional assembly of the wild-type homotetrameric hERG channel complex. We used high-throughput flow cytometry and patch-clamp electrophysiology to assess the trafficking and voltage-dependent activity of hERG channels carrying patient PTC variants that have been corrected by anticodon engineered tRNA. Adenoviral-mediated expression of mutant hERG channels in cultured adult guinea pig cardiomyocytes prolonged action potential durations, and this deleterious effect was corrected upon adenoviral delivery of a human Arg^{UGA} tRNA to restore full-length hERG protein. The results demonstrate mutation-specific, context-agnostic PTC correction and elevate the therapeutic potential of this approach for rare genetic diseases caused by stop codons.

INTRODUCTION

Congenital long-QT syndrome (LQTS) is a cardiac arrhythmia that manifests as a prolongation of the cardiac repolarization phase and is characterized by an extended QT interval on the electrocardiogram. This syndrome is associated with polymorphic ventricular tachycardia, also known as torsades de pointes, and predisposes patients to heart palpitations, syncope, seizures, and sudden cardiac death.[1,2] Approximately 1 in 2,500 people carries a genetic mutation that results in LQTS type 2 (LQTS2). LQTS2 accounts for approximately 30% of the patient population and results from deleterious loss-of-function mutations in the human ether-a-go-go-related gene.[3,4] *KCNH2* (hERG) is located on chromosome 7 and encodes the pore-forming subunit (K_V11.1) of the voltage-gated potassium channel responsible for a major repolarizing current, I_{Kr}, in cardiomyocytes.[5,6] Impairment or loss of hERG function reduces the rate of repolarization and prolongs the cardiac action potential duration, leading to increased propensity for cardiac arrhythmias. Single-nucleotide variants that convert an amino acid-encoding codon into one of the three protein translation stop codons—TAG, TGA, or TAA—represent one such mechanism for impaired hERG function.[7,8] Of these, arginine to TGA mutations are most common, followed by glutamine (TAG/TAA), glutamate (TAG/TAA), tyrosine (TAA/TAG), tryptophan (TGA/TAG), lysine (TGA/TAG), glycine (TGA), leucine (TAA/TAG/TGA), serine (TAA/TAG/TGA), and cysteine (TGA) mutations.[9] The collective burden of protein-terminating variants is not unique to hERG, as nonsense codons represent a common molecular basis for many phenotypically diverse "rare diseases."

There are few therapeutic options for the correction of premature termination codon (PTC) mutations. Powerful gene-editing technologies have the promise of a one-time delivery-and-repair approach, but they are context dependent, and many unique constructs would be required to address the vast number of affected genes. There is promise for small molecules that promote PTC readthrough, but thus far such molecules also diminish encoding fidelity, interact with native stops, and have had poor clinical efficacy.[10–15] By comparison, anticodon-engineered suppressors show promise for the rescue of disease-causing PTCs and have been used to restore function and the mature protein of a PTC-containing mRNA.[12,16–19] Intriguingly, ribosomal profiling data from multiple groups have demonstrated that the *in vitro* or *in vivo* expression of such tRNAs largely spares native stop codons,[20–22] possibly due to the regulated nature of mRNA translation and termination.[23–26] Here, we used anticodon-engineered tRNAs on a panel of

Received 3 February 2023; accepted 14 September 2023;
https://doi.org/10.1016/j.omtn.2023.102032.

[3]These authors contributed equally

Correspondence: Henry M. Colecraft, PhD, Department of Physiology and Cellular Biophysics, Columbia University Medical Center, New York, NY 10032, USA.
E-mail: hc2405@cumc.columbia.edu
Correspondence: Christopher A. Ahern, PhD, Department of Molecular Physiology and Biophysics, University of Iowa Carver College of Medicine, Iowa City, IA, USA.
E-mail: christopher-ahern@uiowa.edu

This is an open access article under the CC BY license (http://creativecommons.org/licenses/by/4.0/).

human disease-causing nonsense mutations in the hERG potassium channel. First, we developed a high-throughput flow cytometry approach in HEK293 cells to: (1) quantify the changes in GFP and hERG protein expression and trafficking due to Arg^{UGA}, Gln^{UAG}, and Trp^{UGA} nonsense mutations, with native mass spectrometry to show insertion of a chosen amino acid at the nonsense mutation site, and (2) determine rescue of full-length hERG protein upon expression of the relevant tRNA for each mutation type. Next, we used patch-clamp electrophysiology to demonstrate tRNA-mediated restoration of hERG whole-cell currents to wild-type (WT) levels. Finally, adenoviral-mediated expression of mutant hERG in cultured adult guinea pig cardiomyocytes yielded prolonged action potential durations, which were normalized by co-expressing Arg^{UGA} tRNA. Together, these results show successful suppression of multiple LQTS2 hERG nonsense mutations in a human cell line and in cardiomyocytes.

RESULTS

Rescue of GFP fluorescence by distinct engineered transfer RNAs

Efficacious anticodon-engineered tRNAs were validated by their capacity to rescue a GFP construct that contained an in-frame TGA stop codon mutation (Figure 1A). We have found that N150TGA effectively truncates GFP expression and is tolerant of a variety of side-chain types. Further, given that Arg-to-TGA PTCs are the most common disease-causing stop-codon variants, we first assessed the encoding fidelity of an Arg^{UGA} tRNA. We used native mass spectrometry to analyze super-folder (sf) GFP N150TGA protein that was rescued by a 4× Arg TCT 3-2-containing plasmid. sfWT GFP or rescued sfGFP-N150TGA protein was purified from HEK cell lysates by exploiting a C-terminal 6×His epitope and analyzed by mass spectrometry (Novatia, Newton, PA, USA) (Figures 1B and 1C). As expected, sfWT GFP gave a mass of 29,141 Da. In the case of the rescued sfGFP-N150TGA, a single peak was identified with a mass of 29,182 Da, within the error of the expected 29,183 Da mass, indicating that a single protein species is produced by Arg insertion at the N150TGA stop site. Consistent with this encoding result, ER-stress pathway markers Bip, Chop, Grp94, Xbp1 splicing, and Trib3 were not upregulated in cells expressing a plasmid containing four copies of an Arg^{UGA} tRNA (Figure S1).

We then utilized a GFP fluorescence reporter assay to determine the efficacy of distinct engineered tRNAs to mediate nonsense suppression of PTCs. Cells expressing WT GFP display a robust green fluorescence signal as reported by flow cytometry analysis (Figures 1D and 1L). By contrast, flow cytometry analysis showed that HEK293 cells expressing sfGFP-N150TAG lacked any intrinsic fluorescence, indicating there is no basal readthrough of the introduced PTC (Figure 1E). Cells co-transfected with sfGFP-N150TAG and GlnUAG tRNA (Figure 1F) displayed robust GFP fluorescence (Figures 1G and 1L), confirming successful suppression of the nonsense mutation and rescued translation of full-length GFP protein. The LQTS2 hERG mutations we investigate in this study include both TAG and TGA nonsense mutations. Therefore, we used the sfGFP-N150TGA construct to evaluate the efficacy of TGA nonsense suppression by anticodon-engineered tRNA species for three distinct amino acids: Arg^{UGA}, Gly^{UGA}, and Trp^{UGA} (Figures 1H–1L). All the tRNAs tested restored GFP fluorescence levels above the background obtained with sfGFP-N150X, albeit with differing efficiencies. The tRNA rank order of nonsense suppression efficiency from this GFP fluorescence assay was $Gln^{UAG} > Arg^{UGA} >> Gly^{UGA} > Trp^{UGA}$ (Figure 1; Table S1). These differences may stem from the tRNA tolerance of anticodon base change or endogenous affinities for translational proteins.[27,28]

Effect of LQTS2 PTC mutations and nonsense suppression on hERG expression and trafficking

We focused our studies on three patient LQTS2 nonsense variants in the hERG C terminus: Q702TAG, R863TGA, and W1001TGA. What impact do these mutations have on hERG protein expression and trafficking to the cell surface, and can any deficiencies in these parameters be effectively rescued by nonsense suppression? To address these questions, we engineered chimeric hERG constructs with mCherry fused to the hERG N terminus and yellow fluorescent protein (YFP) to the C terminus (Figure 2A). This configuration enables mCherry and YFP fluorescence to be used as measures of total protein expression (or stability) and translation of full-length protein, respectively. Further, we introduced a high-affinity bungarotoxin binding site (BBS) into the hERG extracellular S1–S2 loop, which enables selective labeling of the surface population of hERG channels by incubation of non-permeabilized transfected cells with Alexa Fluor 647-conjugated bungarotoxin (BTX-647) (Figure 2A). HEK293 cells expressing WT mCherry-BBS-hERG-YFP displayed strong mCherry, YFP, and BTX-647 fluorescence when examined by confocal microscopy and flow cytometry analyses (Figures 2A–2C and 2M), establishing standards for hERG expression levels, translation of full-length protein, and surface density. Co-expressing WT mCherry-BBS-hERG-YFP with Arg^{UGA} did not have an impact on hERG protein expression, translation, or surface trafficking, indicating tolerance of anticodon-engineered tRNA treatment (Figure S2).

With the tricolor fluorescence assay of hERG expression, translation, and surface density validated, we turned to examining the impact of distinct LQTS2 PTC mutations on these three parameters and their potential to be rescued by suppressor tRNAs. By contrast to WT, cells expressing mCherry-BBS-hERG[Q702X]-YFP displayed 2-fold increases in mCherry and BTX-647 fluorescence, while YFP signal was absent (Figures 2D–2F and 2M; $p < 0.0001$ for mCherry and BTX-647 signal, Dunnett's multiple comparisons test). Thus, this truncated mutant channel shows enhanced protein stability and surface density compared with WT hERG. Co-expressing GlnUAG-engineered tRNA with mCherry-BBS-hERG[Q702X]-YFP recovered YFP fluorescence and normalized both protein stability and surface density to WT levels (Figures 2D–2F and 2M; $p = 0.99$ for mCherry and BTX-647 signal, Dunnett's multiple comparisons test), demonstrating highly effective rescue by nonsense suppression (Table S2).

Cells expressing either mCherry-BBS-hERG[R863X]-YFP or mCherry-BBS-hERG[W1001X]-YFP displayed protein expression levels (mCherry fluorescence) similar to those of WT, but no YFP fluorescence, consistent with production of prematurely truncated

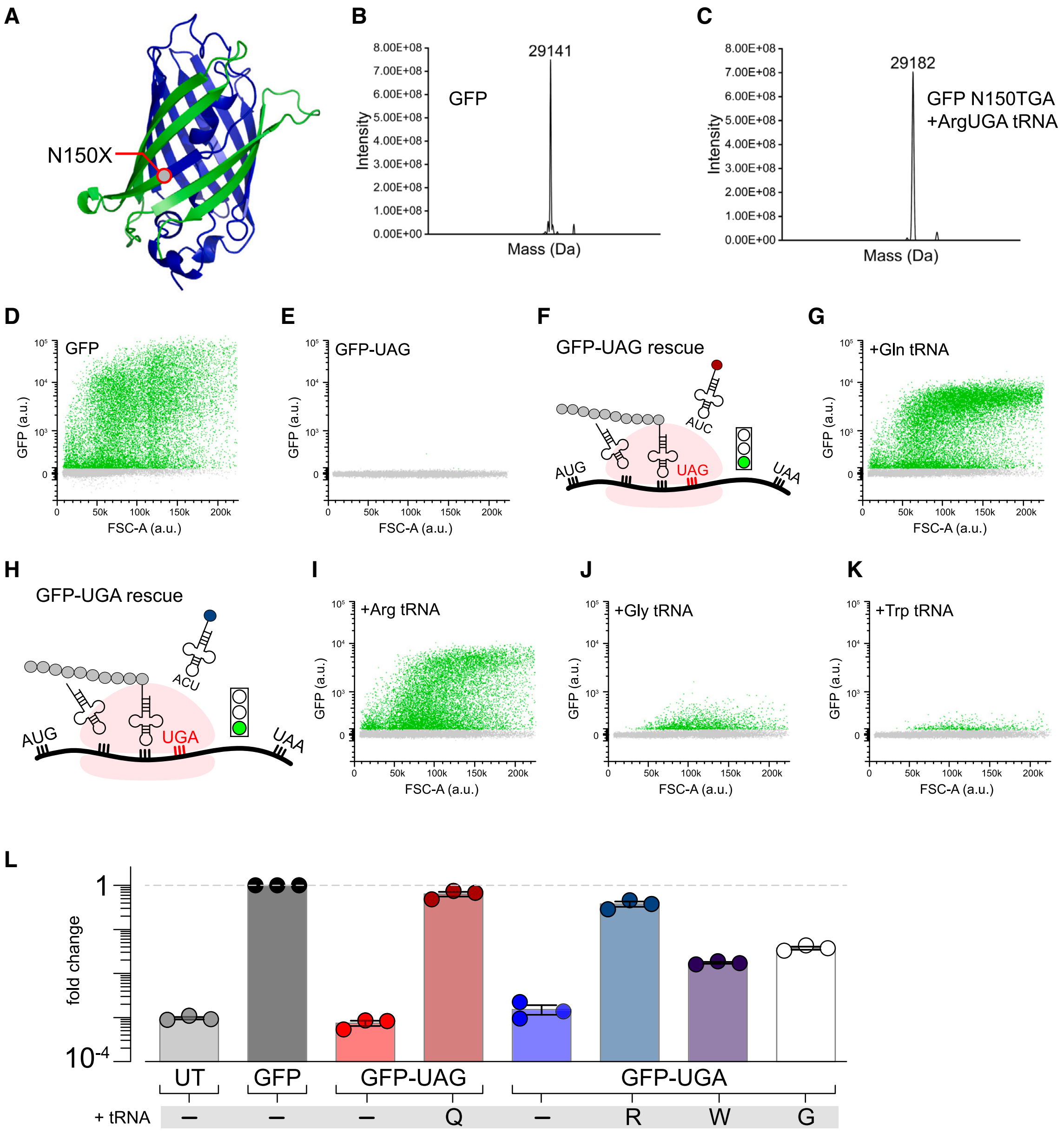

Figure 1. Engineered transfer RNAs (tRNAs) restore full-length and fluorescence of mutant GFP with PTC

(A) Structure of GFP (PDB: 1GFL). Full-length GFP is in green and GFP-UAG (Y151X) is overlaid in blue. (B and C) Native mass spectrometry showing deconvolved masses for GFP and GFP N150X. (D) Flow cytometric analysis of GFP fluorescence where each cell is a single dot. (E) Engineered nonsense mutation in GFP (GFP-UAG) abolishes GFP fluorescence. (F and G) Stop codon suppression with Gln-tRNA partially rescues GFP fluorescence. (H–K) GFP fluorescence of GFP-UGA is also rescued (as shown in H) by (I) Arg-tRNA, (J) Gly-tRNA, and (K) Trp-tRNA and (G) with diminishing efficiencies. (L) Bar graph summarizes population data of normalized GFP fluorescence compared with WT (geometric mean, n = 3).

proteins (Figures 2G–2M). These two mutations differed in that hERG[R863X] displayed a moderately decreased surface density (BTX-647 fluorescence) compared with WT, while this was not observed for hERG[W1001X] (Figures 2G–2M). Co-expressing mCherry-BBS-hERG[R863X]-YFP with Arg^{UGA} tRNA recovered robust YFP fluorescence, normalized channel surface density, and preserved WT levels of protein expression (Figures 2G–2M). By contrast, co-expressing Trp^{UGA} with hERG[W1001X] yielded a relatively poor recovery of YFP fluorescence (Figures 2J–2M), consistent with the suboptimal efficacy of this engineered tRNA in the sfGFP recovery assay (Figures 1K and 1L). Nonsense suppression of hERG [W1001X] was marginally improved by using Gly^{UGA} over Trp^{UGA} as indicated by relatively enhanced YFP fluorescence (Figure 2M), fitting with the moderately higher intrinsic nonsense suppression

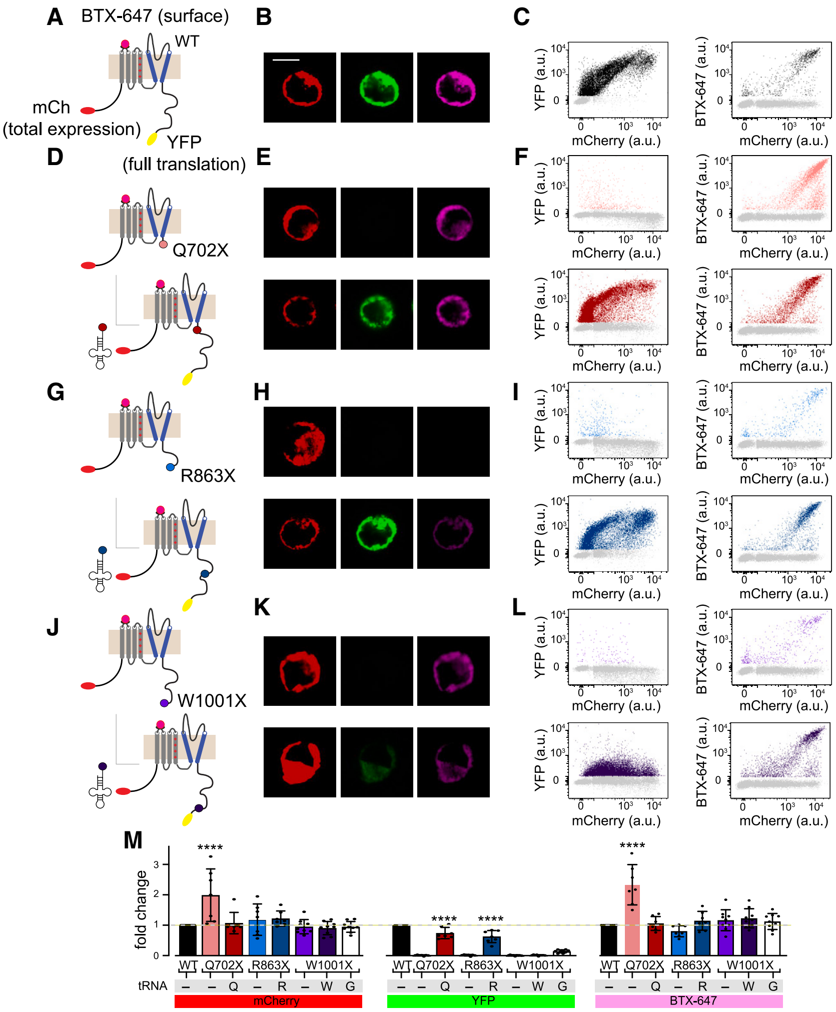

Figure 2. Rescue of patient hERG nonsense mutations with engineered transfer RNA (tRNA) in HEK293 cells
(A) Illustration of engineered WT hERG with mCherry fluorescence tag on the N terminus, YFP on the C terminus, and BTX-647 tagging the extracellular BTX binding site. (B) Confocal images of HEK293 transiently transfected and labeled with BTX-647. Exemplar images show expression (left), full translation of hERG (middle), and trafficking of the channel to the membrane (left). Scale bar, 10 μm. (C) Flow cytometric analysis of population distribution for mCherry, YFP, and BTX-647 fluorescence, where each cell is represented by a single point in a trial. YFP is plotted as a function of mCherry to show the relative proportion of channels fully translated (left). BTX-647 is plotted as a function of mCherry to show relative fraction of hERG trafficked to the plasma membrane (right). (D) Cartoon depiction of hERG with nonsense mutation Q702X. Translation terminates before YFP (top), which is rescued with Gln-tRNA (bottom). (E) Exemplar confocal images show that Q702X mutants are expressed with mCherry fluorescence (top left) and are not fully translated, thus missing YFP fluorescence (top middle), but still traffic to the plasma membrane (top right). Concomitant expression with Gln-tRNA shows mCherry (bottom left), restored YFP (bottom middle), and channel trafficking (bottom right). (F) Flow cytometric analysis of population distribution shows that Q702X truncates the protein, so YFP is not expressed (top left) and may increase channel trafficking (top right). Additional expression of Gln-tRNA restores full-length hERG and YFP fluorescence (bottom left) and reduces channel trafficking to approximately WT levels (bottom right). (G–I) R863X mutant also shows an abolishment of YFP expression with a slight decrease in hERG trafficking. Arg-tRNA rescues YFP expression and hERG trafficking. Format as in (D)–(F). (J–L) W1001X mutant shows loss of YFP with similar hERG trafficking, but Trp-tRNA rescue is less efficient than that of the other tRNAs. Format as in (D)–(F). (M) Population summary of the geometric means of mCherry, YFP, and BTX-647 fluorescence for each of the mutants and tRNAs for each trial (where each trial includes >5,000 cells). Error bars represent standard deviation (mean ± SD). Dunnett's multiple comparisons test was used to determine statistical significance for mCherry and BTX-647 conditions compared with WT (****$p < 0.0001$). Tukey's multiple comparisons test was used for YFP condition to determine statistical significance of rescued protein compared with mutant.

efficacy of this engineered tRNA compared with Trp^{UGA} in the rescued GFP reporter assay (Figure 1).

Nonsense suppressor tRNAs rescue whole-cell currents in LQTS2 mutant hERG channels

Whole-cell patch-clamp electrophysiology was used to determine the functional impact of the distinct LQTS2 hERG mutations on expressed voltage-gated potassium currents and allowed us to directly tested whether any deficiencies were rescued by nonsense suppression (Figure 3). In HEK293 cells expressing WT hERG, step depolarizations (−60 to +40 mV in 20 mV increments) from a holding potential of −80 mV produced robust outward currents. Repolarization to −40 mV yielded characteristic large tail currents due to a fast recovery of the hERG channels from inactivation (Figure 3B). In sharp contrast, cells expressing hERG[Q702X] yielded essentially no currents, consistent with the loss of function expected for a LQTS2 disease-causing mutation (Figure 3D; Table 1). Remarkably, co-expressing hERG[Q702X] with Gln^{UAG} tRNA resulted in the recovery of robust whole-cell K^+ currents that were not significantly different from WT channels (Itail = 136.1 ± 16.8 pA/pF, n = 27 for WT hERG; Itail = 98.3 ± 19.7 pA/pF, n = 25 for hERG[Q702X] + Gln^{UAG} tRNA; p = 0.38, Dunnett's multiple comparisons test) (Figures 3D and 3I).

Similar to hERG[Q702X], cells expressing hERG[R863X] alone displayed essentially no whole-cell K^+ currents; however, co-transfection with Arg^{UGA}-engineered tRNA led to a substantial but, nevertheless, incomplete recovery of current density (Itail = 69.9 ± 17.7 pA/pF, n = 15, p = 0.051, Dunnett's multiple comparisons test) (Figures 3E, 3F, and 3I). In contrast to our observations with hERG[Q702X] and hERG[R863X], cells expressing hERG[W1001X] alone displayed substantial whole-cell current, albeit with a trend toward a reduced tail

current density amplitude compared with WT (Itail = 85.0 ± 10.7 pA/pF, n = 17, p = 0.18, Dunnett's multiple comparisons test) (Figures 3G–3I). Co-transfection of hERG[W1001X] with Trp^{UGA} tRNA did not further increase tail current density. However, Gly^{UGA} tRNA co-expression restored current amplitude to WT levels (Itail = 125.9 ± 25.8 pA/pF, n = 15, p = 0.99, Dunnett's multiple comparisons test) (Figures 3G–3I).

By comparison with WT hERG, currents of mutant channels recovered by nonsense suppression in some cases trended toward displaying a leftward shift in the voltage dependence of activation (Figures 3J–3L), with the V0.5 value for nonsense-suppressed hERG[R863X] reaching statistical difference compared with WT hERG (V0.5 = −7.5 ± 1.5 mV, n = 27 for WT hERG, V0.5 = −18.87 ± 3.4 mV, n = 15 for hERG[R863X] + Arg^{UGA} tRNA, p = 0.002, Dunnett's multiple comparisons test) (Figure 3K; Table 2). By contrast, hERG[Q702X] rescued with GlnUAG had no significant shifts in voltage dependence of activation (V0.5 = −11.2 ± 1.9 mV, n = 21, p = 0.58, Dunnett's multiple comparisons test) (Figure 3J), although the rate of activation and deactivation was depressed at some voltages (Figure S4; Tables S3 and S4). We speculate that the changed kinetics are due to incomplete rescue of full-length proteins by suppressor tRNAs, as evidenced in the flow cytometry data, resulting in some tetrameric channels comprising combinations of full-length and truncated subunits. Tetrameric hERG channels consisting of both rescued and mutant subunits may have differing gating kinetics and trafficking patterns.[29,30]

Nonsense suppression corrects LQTS2 phenotype reconstituted in adult guinea pig ventricular myocytes

We next sought to determine whether the nonsense suppression approach could be effectively applied in actual heart cells, a critical test for the therapeutic potential of this method for heart diseases. In the absence of suitable LQTS2 animal models featuring PTCs in hERG, we developed a cellular LQTS2 model using isolated WT adult guinea pig ventricular myocytes (aGPVMs), which have the contribution of K^+ currents, including hERG (I_{Kr}) and KCNQ1 (I_{Ks}), and a cardiac action potential duration that is comparable to that of humans. After 48–72 h in culture, isolated WT aGPVMs maintained the rod-shaped architecture of healthy adult mammalian cardiomyocytes and displayed no mCherry or YFP fluorescence (Figures 4A and 4B). Current clamp recordings of these aGPVMs paced at 0.5 Hz revealed archetypal cardiac action potentials (APD80 = 535.5 ± 91.9 ms, n = 9) (Figures 4C and 4J). Contemporaneously cultured aGPVMs infected with adenovirus encoding WT mCherry-BBS-hERG-YFP (infections carried out 2 h after isolation) displayed both mCherry and YFP fluorescence (Figures 4D and 4E) and did not significantly affect action potential duration (APD80 = 423.5 ± 45.6, n = 12, p = 0.41, one-way ANOVA and Dunnett's multiple comparisons test) compared with uninfected cells (Figures 4F and 4J). Patch-clamp electrophysiology in HEK cells indicated that the addition of the fluorophores and BBS to hERG had no effect on current density, although there was a leftward shift in the current-voltage relationship (Figure S3, Student's t test, p = 0.25). aGPVMs infected with mCherry-BBS-hERG[R863X]-YFP adenovirus displayed mCherry but not YFP fluorescence, confirming expression of a truncated hERG protein in these cells (Figures 4G and 4H). Moreover, these cells displayed a significantly prolonged action potential compared with control (APD80 = 699.8 ± 44.0 ms, n = 13, p = 0.0023, one-way ANOVA and Dunnett's multiple comparisons test), suggesting a dominant-negative effect of the truncated hERG construct and mimicking the autosomal dominant phenotype of LQTS2. Importantly, co-expressing Arg^{UGA} tRNA with mCherry-BBS-hERG[R863X]-YFP rescued translation of full-length hERG protein, as indicated by the emergence of YFP fluorescence (Figures 4G and 4H) and normalized action potential duration (APD80 = 444.7 ± 48.5 ms, n = 14, p = 0.98, one way ANOVA and Dunnett's multiple comparisons test) (Figures 4I and 4J, also summarized in Table S5). Altogether, these results demonstrate the efficacy of suppressor tRNAs in rescuing pathologies associated with nonsense mutations in adult cardiac myocytes.

DISCUSSION

In this work, we have investigated the capacity of anticodon-engineered tRNAs to rescue three PTC variants (Q702X, R863X, and W1001X) in the intracellular C terminus of hERG that cause LQTS2. The hERG C terminus (residues 638–1,159) is divided into a proximal region, containing a C linker (residues 638–764) and cyclic nucleotide binding homology domain (CNBD; residues 765–837), and a distal C terminus (residues 838–1,159).[31,32] The distal C terminus contains an RXR signal important for channel trafficking (residues 1,005–1,007) and a tetramerization coiled-coil domain (residues 1,036–1,074) essential for channel assembly.[33,34] We found that cells expressing either Q702X and R863X yielded no currents, while homotetrameric W1001X channels yielded whole-cell currents, albeit with a trend toward reduced amplitude compared with WT channels. These results are in broad agreement with previous studies on R863X and W1001X channels.[35,36] Surprisingly, we found that Q702X showed enhanced protein stability and surface density compared with WT, despite not displaying any currents. The difference in protein stability and surface density between Q702X and R863X suggests there may be determinants between residues 702 and 863 that negatively regulate these processes.

Of the four engineered tRNA types tested, we found that Arg^{UGA} tRNA and Gln^{UAG} tRNA displayed higher rescue profiles in both a GFP reporter assay and mutant hERG channels, compared with Gly^{UGA} and Trp^{UGA}. The higher rescue efficacy of Arg^{UGA} tRNA and Gln^{UAG} tRNA is fortuitous given that these PTC types are the more common variants. Moreover, the similar rank order in rescue efficacy of the four tRNAs between GFP and hERG suggests that the context of the PTC has minimal impact on its capacity to be corrected by nonsense suppression. Trp^{UGA} tRNA rescued hERG W1001TGA channels less efficiently, but this low suppressor function was also seen in GFP. Thus, this level of W1001TGA rescue is likely due to the poor performance of the Trp^{UGA} tRNA. Consistent with this possibility, we found that the hERG W1001TGA PTC could be well corrected by Gly^{UGA} tRNA. This suggests that in some cases, such as W1001TGA, other tRNA types might be used for correction. We have previously identified

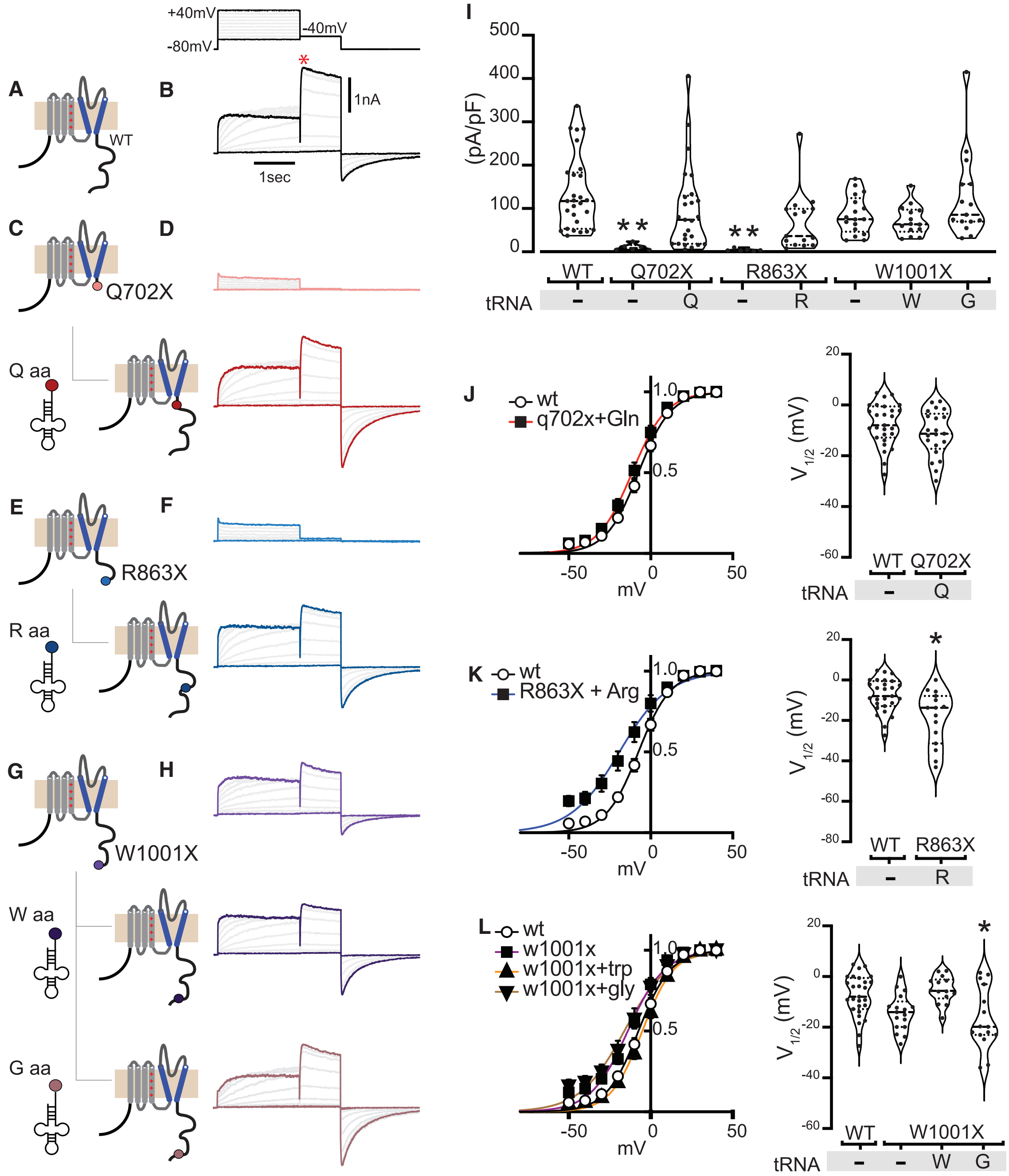

Figure 3. Mutant hERG co-expressed with an engineered transfer RNA (tRNA) is functionally similar to WT channels

(A) Cartoon depiction of WT hERG transfected into HEK293. (B) Exemplar traces of WT hERG with a voltage-step protocol where the cell is depolarized from −80 mV to a step voltage ranging from −60 to +40 mV in 20 mV increments for 2 s and then back to +40 mV for 1 s with a repetition interval of 2 s. (C) HEK293 cells were transfected with the Q702X mutant alone (top) or with Gln-tRNA (bottom). (D) Exemplar current traces for Q702X voltage-step protocol showing that mutant channels to not exhibit much outward current (top). Gln-tRNA rescues potassium currents to levels comparable to those of WT (bottom). (E and F) R863X mutants also show reduced potassium currents, which are restored with Arg-tRNA. Format as in (C) and (D). (G and H) W1001X mutants show less reduction in potassium currents, and the addition of Trp-tRNA does not appear to markedly increase current size. Format as in (C) and (D). (I) Violin plots of population data for each of the conditions, where each point represents a single cell. Dunnett's multiple comparisons test was used to determine statistical significance from WT ($p < 0.05$). (J) Conductance-voltage curve (mean ± SEM) for Q702X with tRNA condition (left) and violin plots of population data, where each point represents the $V_{0.5}$ of a single cell. A Dunnett's multiple comparisons test was used for statistical significance (*$p < 0.05$. **$p < 0.005$) (right). (K) Conductance-voltage curve (mean ± SEM) for R863X with tRNA condition (left) and violin plots of population data, where each point represents the $V_{0.5}$ of a single cell. A Dunnett's multiple comparisons test was used for statistical significance (*$p < 0.05$. **$p < 0.005$) (right). (L) Conductance-voltage curve (mean ± SEM) for W1001X with tRNA condition (left) and violin plots of population data, where each point represents the $V_{0.5}$ of a single cell. A Dunnett's multiple comparisons test was used for statistical significance (*$p < 0.05$. **$p < 0.005$) (right).

Table 1. Current density of rescued hERG

Condition (n)	Current density (pA/pF)
WT hERG (27)	136.1 ± 16.79
Q702X (6)	10.48 ± 3.06*
Q702x + Gln-tRNA (25)	98.27 ± 19.72
R863X (6)	4.48 ± 1.26*
R863X + Arg-tRNA (15)	69.88 ± 17.75
W1001X (17)	85.05 ± 10.73
W1001X + Trp-tRNA (14)	72.43 ± 9.32
W1001X + Gly-tRNA (15)	125.9 ± 25.8

Values are presented as the mean ± SEM. Statistical significance was set at $p < 0.05$ and is marked with an asterisk. The data in this table were used to generate Figure 3.

Table 2. Conductance-voltage (GV) relationships of rescued hERG potassium channels

	GV	
Condition (n)	$V_{1/2}$ (mV)	k (slope)
WT hERG (27)	−7.5 ± 1.5	8.7 ± 0.3
Q702x + Gln-tRNA (21)	−11.2 ± 1.9	8.9 ± 0.3
R863X + Arg-tRNA (15)	−18.8 ± 3.4*	12.6 ± 0.8*
W1001X (17)	−14.2 ± 1.7	10.7 ± 0.3
W1001X + Trp-tRNA (14)	−5.1 ± 1.4	9.1 ± 0.3
W1001X + Gly-tRNA (15)	−16.7 ± 3.1*	12.2 ± 1.1*

Values are presented as the mean ± SEM. Statistical significance was set at $p < 0.05$ and is marked with an asterisk. The data in this table were used to generate Figure 3.

suppressor tRNAs for many human-disease-causing PTCs, including hydrophobic leucine and isoleucine residues.[20]

PTC readthrough and gene therapy strategies that are currently under development face several ongoing challenges. Small-molecule approaches promote indiscriminate missense encoding at PTCs,[37] yet many proteins, such as hERG, are poorly tolerant of such changes.[38,39] Notably, engineered tRNAs, for those that have been tested, encode the intended amino acid, although further examination is merited for many tRNA classes.[21] Moreover, there remains the possibility of assessing tolerance for a specific amino acid at a given site, as we found with W1001TGA. Nucleotide editing methods, such as CRISPR and base editing, modify germline DNA or mRNA, yet the diverse contexts of PTC mutations and overall low patient populations for each mutation are challenges for each gene-editing construct. In addition, CRISPR faces ongoing optimization efforts related to off-site editing and immunoresponse to bacterial CRISPR components.[40,41] Adeno-associated virus (AAV) is a promising delivery method, but has a limited payload (~4 kb), which rules out large targets, including many ion channels, transporters, and cytoskeletal proteins.[42–44] Further, many such targets are tightly regulated at the mRNA and protein levels, suggesting that simple "overexpression" gene therapies or interventions that activate the endogenous promotor of the unaffected allele may lead to unregulated gene expression. Suppressor tRNAs repair PTCs post-transcriptionally, at the level of mRNA, which suggests that WT protein levels can be restored, but not exceeded, and native regulatory processes will remain unaffected. Indeed, such tRNA-based approaches may be one of the only means to biologically repair a somatic PTC in a mutation-specific, gene and tissue-agnostic manner. Further, given the compact size of tRNA (~75 bp), it is possible to simultaneously encode multiple copies of a given isodecoder within a single AAV payload. We also demonstrate the efficacy in packaging four copies of an Arg^{UGA} tRNA in Ad5 viral particles, and these are highly effective in correcting the cardiac action potential in hERG PTC mutants expressing freshly dissociated myocytes. A limitation of the study is that hERG currents were not directly measured in the cardiomyocytes. Until an animal model is generated, further proof of tRNA therapeutic qualities for LQTS2 is not possible. In the case of LQTS, there are few treatment options available for patients outside of lifestyle changes, such as avoiding strenuous exercise and taking beta blockers.[45] Patients non-receptive to first-line treatments will require more invasive, costly, and disruptive procedures, such as heart surgeries for left cardiac sympathetic denervation or implantation of defibrillators. Our data suggest that engineered suppressor tRNAs may be an option for the correction of post-mitotic cells harboring genes with PTCs.

MATERIALS AND METHODS

Plasmid constructs and mutagenesis

WT hERG1a constructs were generous gifts from Dr. Alfred George (Northwestern University) and Dr. Gail Robertson (University of Wisconsin). HERG1a-YFP was generated by using overlap extension polymerase chain reaction (PCR) to fuse YFP in frame to the C terminus of hERG1a, as described previously.[39] Sequence for a BBS (13 amino acid residues) was placed between residues Thr436 and Glu437 in the hERG1a extracellular S1–S2 loop using the QuikChange Lightning site-directed mutagenesis kit (Stratagene) to generate BBS-hERG1a-YFP. The BBS-hERG1a-YFP cassette was excised with BamH1/KpnI and placed in frame to the C terminus of mCherry in the pcDNA3 plasmid to generate mCherry-BBS-hERG1a-YFP. Premature stop codon mutations in hERG1a (Q702TAG, R863TGA, and W1001) were introduced by site-directed mutagenesis or gene block synthesis and ligation.

HEK293 cell culture and transient transfection

Low-passage HEK293 cells were maintained in high-glucose Dulbecco's minimum essential medium (DMEM) supplemented with 10% fetal bovine serum (FBS), 1% penicillin-streptomycin, and 1% L-glutamine. Cells were incubated in a 5% CO_2 humidified incubator at 37°C. Transient transfections for hERG flow cytometry experiments used the calcium phosphate precipitation method. Briefly, plasmid DNA for WT or mutant mCherry-BBS-hERG1a-YFP (1 μg) was mixed with tRNA (1 μg) and T antigen (500 ng) with 7.75 μL of 2 M $CaCl_2$ and sterile deionized water to a final volume of 62.5 μL. The mixture was added dropwise to 62.5 μL of 2× HEPES-buffered saline containing HEPES, 50 mM; NaCl, 280 mM; and Na_2HPO_4, 1.5 mM (pH 7.09). The resulting DNA-calcium phosphate mixture was incubated for 20–30 min at room temperature and added dropwise to 60%–80%

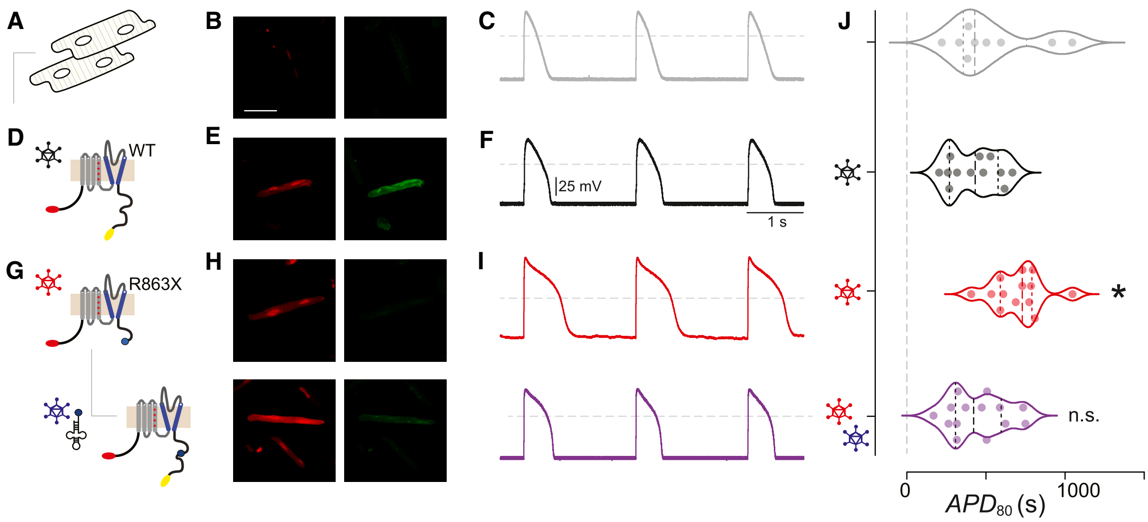

Figure 4. Engineered transfer RNA (tRNA) rescued prolonged action potentials in aGPVMs expressing mutant hERG

(A) Action potentials were measured from isolated aGPVMs. (B) Confocal images of uninfected aGPVMs. Exemplar images show no mCherry (left) or YFP (right) expression. Scale bar, 50 μm. (C) Exemplar trace of action potentials measured from uninfected aGPVMs. (D) Cartoon depiction of adenovirus delivering WT hERG construct to aGPVM. (E) Confocal images show robust expression of mCherry (left) and YFP (right) for WT hERG. (F) Action potential of aGPVMs infected with WT hERG shows not much difference compared with uninfected aGPVMs. (G) Cartoon depiction of adenovirus delivering mutant R863X hERG (top) and rescue with another adenovirus carrying Arg-tRNA (bottom). (H) Confocal images show mCherry expression for both mutant (top left) and rescued hERG (bottom left). YFP is not expressed in mutant hERG (top right) but is rescued with Arg-tRNA (bottom right). (I) R863X-infected aGPVMs show prolonged action potentials (top), which is rescued with Arg-tRNA expression (bottom). (J) Violin plot of population data for each of the viral conditions, where each point represents average APD_{80} collected from one cell (**p = 0.0025 compared with WT, one-way ANOVA and Dunnett's multiple comparisons test).

confluent HEK293 cells cultured in 12 well tissue culture dishes. Cells were washed with Ca^{2+}-free phosphate-buffered saline after 4–6 h and maintained in supplemented DMEM. Transient transfections of hERG for patch-clamp recordings were done using FuGENE HD transfection reagent (Promega) according to the manufacturer's instructions.

Adult guinea pig ventricular myocyte isolation and cell culture

Cardiomyocytes were isolated from whole hearts of Hartley strain guinea pigs (Charles River) in accordance with the guidelines of the Columbia University Animal Care and Use Committee. Animals were anesthetized with 3%–5% isoflurane and maintained on the anesthesia plane with 1%–3% isoflurane in accordance with IACUC guidelines (protocol no. AC-AABG9557). Hearts were excised under deep anesthesia, with depth of anesthesia confirmed by loss of righting reflex and toe-pinch response. Excised hearts were perfused on a Langendorff perfusion apparatus first with KH solution (118 mM NaCl, 4.8 mM KCl, 1 mM $CaCl_2$, 25 mM HEPES, 1.25 mM K_2HPO_4, 1.25 mM $MgSO_4$, 11 mM glucose, and 0.02 mM EGTA [pH 7.4]), followed by KH solution without calcium. Hearts were then enzymatically digested with 0.3 mg/mL collagenase type 4 (Worthington), 0.08 mg/mL protease, and 0.05% BSA, followed by KH buffer without calcium. Finally, 40 mL of high-K^+ solution was perfused through the heart: 120 mM potassium glutamate, 25 mM KCl, 10 mM HEPES, 1 mM $MgCl_2$, and 0.02 mM EGTA (pH 7.4). Chunks of heart were gently triturated in high-K^+ solution to yield single rod-shaped myocytes. Dissociated cardiomyocytes were subsequently plated on glass coverslips coated with 15 μg/mL laminin (Corning) and cultured in Medium 199 (Life Technologies) supplemented with 10 mM HEPES (Gibco), 1× MEM non-essential amino acids (Gibco), 2 mM L-glutamine (Gibco), 20 mM D-glucose (Sigma Aldrich), 1% (v/v) penicillin-streptomycin-glutamine (Fisher Scientific), 0.02 mg/mL vitamin B-12 (Sigma Aldrich), and 5% (v/v) FBS (Life Technologies) to promote attachment to dishes. After 2 h, the culture medium was switched to Medium 199 with 1% (v/v) serum, but otherwise supplemented as described above. Adenovirus vectors expressing WT or mutant hERG ± engineered tRNA were also added at this time for specified conditions. Cultures were maintained in humidified incubators at 37°C and 5% CO_2 and used for electrophysiological recordings after 48 h of culture.

Measuring ER stress

HEK293T cells were plated in six well plates using DMEM (10% FBS, 1% Pen-Strep, 1% L-Glu) and transfected at 70% confluency with 0.2 μg of 4× Arg TGA TCT 3-2 GFP/4× scrambled tRNA sequence GFP as triplicates using PolyJet transfection reagent. The medium was changed after 12 h. Tunicamycin (1:1,000) was added 8 h prior to harvesting. Cells were harvested ~42 h post-transfection using Trizol. RNA was isolated using Trizol (Thermo Fisher, USA) following the manufacturer's protocol, and RNA concentrations were evaluated using the Qubit RNA Broad Range kit (Invitrogen, USA). Four hundred nanograms of RNA from each group was used for reverse transcription using the PrimeScript RT Master Mix (Takara, USA). PCRs were performed using the iTaq Universal SYBR Green Supermix (Bio-Rad, USA). Information on primers is listed below. Gene

expression was normalized against the average of two loading controls (*Btf3* and *Ppia*).

The qRT-PCR primers were as follows:

Btf3 forward, CCAGTTACAAGAAAGGCTGCT; reverse, CTTCA ACAGCTTGTCCGCT

Ppia forward, AGCACTGGAGAGAAAGGATT; reverse, ATTAT GGCGTGTAAAGTCACCA

Chop forward, CTGCCTTTCACCTTGGAGAC; reverse, CGTTT CCTGGGGATGAGATA

Bip forward, CATGGTTCTCACTAAAAT; reverse, GCTGGTA CAGTAACAACTG

Gadd34 forward, GAGATTCCTCTAAAAGCTCGG; reverse, CA GGGACCTCGACGGCAGC

Grp94 forward, AATAGAAAGAATGCTTCGCC; reverse, TCT TCAGGCTCTTCTTCTGG

Xbp1 (spliced) forward, GAGTCCGCAGCAGGTG; reverse, GTG TCAGAGTCCATGGGA

Mass spectrometry

HEK293T cells were plated in 10 cm dishes using DMEM (10% FBS, 1% Pen-Strep, 1% L-Glu) and transfected at 70% confluency with 5 μg of 4× Arg TGA TCT 3-2 TdTom and sfGFPTGA-His using PolyJet transfection reagent. The medium was changed after 12 h. Cells were lysed using Rippa lysis buffer (Thermo Scientific) with protease inhibitor 24 h post-transfection. Supernatants were collected and run through a poly-prep chromatography column (Bio-Rad). Samples were processed by Novatia for mass spectrometry.

Flow cytometry

Fluorescence detection of WT and mutant hERG1a total protein expression, nonsense suppression rescue by engineered tRNA, and relative surface density by flow cytometry was accomplished as previously described. Briefly, 48 h after transfection, cells cultured in 12 well plates were washed with ice-cold PBS containing Ca^{2+} and Mg^{2+} (0.9 mM $CaCl_2$, 0.49 mM $MgCl_2$ [pH 7.4]) and then incubated for 30 min in blocking medium (DMEM with 3% BSA) at 4°C. The cells were then incubated with 1 μM Alexa Fluor 647-conjugated α-bungarotoxin (BTX-647; Life Technologies) in DMEM with 3% BSA on a rocker at 4°C for 1 h, followed by three washes with PBS (containing Ca^{2+} and Mg^{2+}). Cells were gently harvested in Ca^{2+}-free PBS and assayed by flow cytometry using a BD LSRII cell analyzer (BD Biosciences, San Jose, CA, USA). Fluorophores were excited with distinct excitation wavelengths (YFP, 488 nm; mCherry, 561 nm; Alexa Fluor 647, 633 nm).

Patch-clamp electrophysiology

HEK293T cells were transfected with equal amounts of hERG and tRNA cDNA along with 500 ng of GFP cDNA. In rescue conditions, GFP had a nonsense mutation, which allowed for tRNA-positive selection of cells. Transfected cells were incubated at 32°C for 48 h before use. Whole-cell currents were recorded at room temperature using a Multiclamp 700B patch-clamp amplifier (Axon Instruments) controlled by pClamp software. Analysis was done using Clampfit. The internal solution contained 130 mmol/L KCl, 1 mmol/L $MgCl_2$, 0.4 mmol/L GTP, 5 mmol/L EGTA, 5 mmol/L K_2ATP, and 10 mmol/L HEPES (pH 7.2). The external solution contained 137 mmol/L NaCl, 4 mmol/L KCl, 1.8 mmol/L $CaCl_2$, 1 mmol/L $MgCl_2$, 10 mmol/L glucose, and 10 mmol/L HEPES (pH 7.4). When filled with internal solution, the pipette resistance was typically 1.5–2.5 MW. Current recordings were generated by step depolarizations (−50 to +40 mV), from a holding potential of −80 mV in 10 mV increments for 2 s, followed by a repolarizing step to −40 mV for 1 s and a 2 s repetition interval. Current-clamp recordings of cardiomyocytes were performed 48 h after adenoviral infection. Recordings for all experimental conditions were interleaved and had time-matched controls from the same cardiomyocyte cultures. Internal solution contained K glutamate, 130 mM; KCl, 9 mM; NaCl, 10 mM; $MgCl_2$, 0.5 mM; EGTA, 0.5 mM; MgATP, 4 mM; HEPES, 10 mM (adjusted to pH 7.3 with KOH). The external solution contained NaCl, 135 mM; KCl, 5.4 mM; $CaCl_2$, 1.8 mM; $MgCl_2$, 0.33 mM; NaH_2PO_4, 0.33 mM; HEPES, 5 mM; glucose, 5 mM (pH 7.4). The time from upstroke to 80% repolarization (APD_{80}) was measured with custom MATLAB (MathWorks) software and used as a metric for comparing physiological output between peptide-treated and untreated. Currents were filtered at 5 kHz and sampled at 25 kHz with a P/8 leak subtraction.

DATA AND CODE AVAILABILITY

All data from this study are included in this article, the associated source data file, and the supplemental information.

SUPPLEMENTAL INFORMATION

Supplemental information can be found online at https://doi.org/10.1016/j.omtn.2023.102032.

ACKNOWLEDGMENTS

This work was supported by grants from the Roy J. Carver Charitable Trust (to C.A.A.) and NIH RO1 HL142111 (to H.M.C.). The authors thank Columbia University in the City of New York and the Rabi Scholars Program for their generous support of V.G.B. These studies used the resources of the Herbert Irving Comprehensive Cancer Center Confocal and Specialized Microscopy Shared Resource, funded in part through NIH/NCI Cancer Center Support Grant P30CA013696.

AUTHOR CONTRIBUTIONS

Conceptualization, V.G.B., J.N., H.M.C., and C.A.A.; methodology, V.G.B.; investigation, V.G.B., J.N., and P.C.; visualization, V.G.B., J.N., and P.C.; writing – original draft, V.G.B. and J.N.; writing – reviewing & editing, V.G.B., H.M.C., and C.A.A.; supervision, H.M.C. and C.A.A.; funding acquisition, H.M.C. and C.A.A.

DECLARATION OF INTERESTS

C.A.A. is a scientific co-founder of hC Biosciences and holds stock in Tevard Bioscience.

REFERENCES

1. Priori, S.G., Wilde, A.A., Horie, M., Cho, Y., Behr, E.R., Berul, C., Blom, N., Brugada, J., Chiang, C.E., Huikuri, H., et al. (2013). HRS/EHRA/APHRS expert consensus statement on the diagnosis and management of patients with inherited primary arrhythmia syndromes: document endorsed by HRS, EHRA, and APHRS in May 2013 and by ACCF, AHA, PACES, and AEPC in June 2013. Heart Rhythm *10*, 1932–1963. https://doi.org/10.1016/j.hrthm.2013.05.014.

2. Wilde, A.A.M., Amin, A.S., and Postema, P.G. (2022). Diagnosis, management and therapeutic strategies for congenital long QT syndrome. Heart *1085*, 332–338. https://doi.org/10.1136/heartjnl-2020-318259.

3. Wallace, E., Howard, L., Liu, M., O'Brien, T., Ward, D., Shen, S., and Prendiville, T. (2019). Long QT Syndrome Genetics and Future Perspective. Pediatr Cardiol *40*, 1419–1430. https://doi.org/10.1007/s00246-019-02151-x.

4. Kapplinger, J.D., Tester, D.J., Salisbury, B.A., Carr, J.L., Harris-Kerr, C., Pollevick, G.D., Wilde, A.A.M., and Ackerman, M.J. (2009). Spectrum and prevalence of mutations from the first 2,500 consecutive unrelated patients referred for the FAMILION long QT syndrome genetic test. Heart Rhythm *6*, 1297–1303. https://doi.org/10.1016/j.hrthm.2009.05.021.

5. Curran, M.E., Splawski, I., Timothy, K.W., Vincent, G.M., Green, E.D., and Keating, M.T. (1995). A molecular basis for cardiac arrhythmia: HERG mutations cause long QT syndrome. Cell *80*, 795–803.

6. Vandenberg, J.I., Perry, M.D., Perrin, M.J., Mann, S.A., Ke, Y., and Hill, A.P. (2012). hERG K(+) channels: structure, function, and clinical significance. Physiol. Rev. *92*, 1393–1478. https://doi.org/10.1152/physrev.00036.2011.

7. Bhuiyan, Z.A., Momenah, T.S., Gong, Q., Amin, A.S., Ghamdi, S.A., Carvalho, J.S., Homfray, T., Mannens, M.M.A.M., Zhou, Z., and Wilde, A.A.M. (2008). Recurrent intrauterine fetal loss due to near absence of HERG: clinical and functional characterization of a homozygous nonsense HERG Q1070X mutation. Heart Rhythm *5*, 553–561. https://doi.org/10.1016/j.hrthm.2008.01.020.

8. Ko, Y.L., Tai, D.Y., Chen, S.A., Lee-Chen, G.J., Chu, C.H., and Lin, M.W. (2001). Linkage and mutation analysis in two Taiwanese families with long QT syndrome. J. Formos. Med. Assoc. *100*, 767–771.

9. Mort, M., Ivanov, D., Cooper, D.N., and Chuzhanova, N.A. (2008). A meta-analysis of nonsense mutations causing human genetic disease. Hum. Mutat. *29*, 1037–1047. https://doi.org/10.1002/humu.20763.

10. Kerem, E., Konstan, M.W., De Boeck, K., Accurso, F.J., Sermet-Gaudelus, I., Wilschanski, M., Elborn, J.S., Melotti, P., Bronsveld, I., Fajac, I., et al. (2014). Ataluren for the treatment of nonsense-mutation cystic fibrosis: a randomised, double-blind, placebo-controlled phase 3 trial. Lancet Respir. Med. *2*, 539–547. https://doi.org/10.1016/S2213-2600(14)70100-6.

11. Chen, J., Thrasher, K., Fu, L., Wang, W., Aghamohammadzadeh, S., Wen, H., Tang, L., Keeling, K.M., Falk Libby, E., Bedwell, D.M., and Rowe, S.M. (2023). The synthetic aminoglycoside ELX-02 induces readthrough of G550X-CFTR producing superfunctional protein that can be further enhanced by CFTR modulators. Am. J. Physiol. Lung Cell Mol. Physiol. *324*, L756–L770. https://doi.org/10.1152/ajplung.00038.2023.

12. Keeling, K.M., Xue, X., Gunn, G., and Bedwell, D.M. (2014). Therapeutics based on stop codon readthrough. Annu. Rev. Genomics Hum. Genet. *15*, 371–394. https://doi.org/10.1146/annurev-genom-091212-153527.

13. Sharma, J., Du, M., Wong, E., Mutyam, V., Li, Y., Chen, J., Wangen, J., Thrasher, K., Fu, L., Peng, N., et al. (2021). A small molecule that induces translational readthrough of CFTR nonsense mutations by eRF1 depletion. Nat. Commun. *12*, 4358. https://doi.org/10.1038/s41467-021-24575-x.

14. Wangen, J.R., and Green, R. (2020). Stop codon context influences genome-wide stimulation of termination codon readthrough by aminoglycosides. Elife *9*, e52611. https://doi.org/10.7554/eLife.52611.

15. Wohlgemuth, I., Garofalo, R., Samatova, E., Günenç, A.N., Lenz, C., Urlaub, H., and Rodnina, M.V. (2021). Translation error clusters induced by aminoglycoside antibiotics. Nat. Commun. *12*, 1830. https://doi.org/10.1038/s41467-021-21942-6.

16. Temple, G.F., Dozy, A.M., Roy, K.L., and Kan, Y.W. (1982). Construction of a functional human suppressor tRNA gene: an approach to gene therapy for beta-thalassaemia. Nature *296*, 537–540.

17. Buvoli, M., Buvoli, A., and Leinwand, L.A. (2000). Suppression of nonsense mutations in cell culture and mice by multimerized suppressor tRNA genes. Mol. Cell Biol. *20*, 3116–3124.

18. Bordeira-Carriço, R., Ferreira, D., Mateus, D.D., Pinheiro, H., Pêgo, A.P., Santos, M.A.S., and Oliveira, C. (2014). Rescue of wild-type E-cadherin expression from nonsense-mutated cancer cells by a suppressor-tRNA. Eur. J. Hum. Genet. *22*, 1085–1092. https://doi.org/10.1038/ejhg.2013.292.

19. Ko, W., Porter, J.J., Sipple, M.T., Edwards, K.M., and Lueck, J.D. (2022). Efficient suppression of endogenous CFTR nonsense mutations using anticodon-engineered transfer RNAs. Mol. Ther. Nucleic Acids *28*, 685–701. https://doi.org/10.1016/j.omtn.2022.04.033.

20. Lueck, J.D., Yoon, J.S., Perales-Puchalt, A., Mackey, A.L., Infield, D.T., Behlke, M.A., Pope, M.R., Weiner, D.B., Skach, W.R., McCray, P.B., Jr., and Ahern, C.A. (2019). Engineered transfer RNAs for suppression of premature termination codons. Nat. Commun. *10*, 822. https://doi.org/10.1038/s41467-019-08329-4.

21. Wang, J., Zhang, Y., Mendonca, C.A., Yukselen, O., Muneeruddin, K., Ren, L., Liang, J., Zhou, C., Xie, J., Li, J., et al. (2022). AAV-delivered suppressor tRNA overcomes a nonsense mutation in mice. Nature *604*, 343–348. https://doi.org/10.1038/s41586-022-04533-3.

22. Albers, S., Allen, E.C., Bharti, N., Davyt, M., Joshi, D., Perez-Garcia, C.G., Santos, L., Mukthavaram, R., Delgado-Toscano, M.A., Molina, B., et al. (2023). Engineered tRNAs suppress nonsense mutations in cells and *in vivo*. Nature *618*, 842–848. https://doi.org/10.1038/s41586-023-06133-1.

23. Korostelev, A.A. (2022). The Structural Dynamics of Translation. Annu. Rev. Biochem. *91*, 245–267. https://doi.org/10.1146/annurev-biochem-071921-122857.

24. Loveland, A.B., Demo, G., and Korostelev, A.A. (2020). Cryo-EM of elongating ribosome with EF-Tu*GTP elucidates tRNA proofreading. Nature *584*, 640–645. https://doi.org/10.1038/s41586-020-2447-x.

25. Holm, M., Natchiar, S.K., Rundlet, E.J., Myasnikov, A.G., Watson, Z.L., Altman, R.B., Wang, H.Y., Taunton, J., and Blanchard, S.C. (2023). mRNA decoding in human is kinetically and structurally distinct from bacteria. Nature *617*, 200–207. https://doi.org/10.1038/s41586-023-05908-w.

26. Ibba, M., and Söll, D. (1999). Quality control mechanisms during translation. Science *286*, 1893–1897. https://doi.org/10.1126/science.286.5446.1893.

27. Porat, J., Kothe, U., and Bayfield, M.A. (2021). Revisiting tRNA chaperones: New players in an ancient game. RNA *27*, 543–559. https://doi.org/10.1261/rna.078428.120.

28. Yarus, M., Cline, S., Raftery, L., Wier, P., and Bradley, D. (1986). The translational efficiency of tRNA is a property of the anticodon arm. J. Biol. Chem. *261*, 10496–10505.

29. Zhou, Z., Gong, Q., Epstein, M.L., and January, C.T. (1998). HERG channel dysfunction in human long QT syndrome. Intracellular transport and functional defects. J. Biol. Chem. *273*, 21061–21066. https://doi.org/10.1074/jbc.273.33.21061.

30. Zhao, J.T., Hill, A.P., Varghese, A., Cooper, A.A., Swan, H., Laitinen-Forsblom, P.J., Rees, M.I., Skinner, J.R., Campbell, T.J., and Vandenberg, J.I. (2009). Not all hERG pore domain mutations have a severe phenotype: G584S has an inactivation gating defect with mild phenotype compared to G572S, which has a dominant negative trafficking defect and a severe phenotype. J. Cardiovasc. Electrophysiol. *20*, 923–930. https://doi.org/10.1111/j.1540-8167.2009.01468.x.

31. Barros, F., Domínguez, P., and de la Peña, P. (2012). Cytoplasmic domains and voltage-dependent potassium channel gating. Front. Pharmacol. *3*, 49. https://doi.org/10.3389/fphar.2012.00049.

32. Brelidze, T.I., Carlson, A.E., Sankaran, B., and Zagotta, W.N. (2012). Structure of the carboxy-terminal region of a KCNH channel. Nature *481*, 530–533. https://doi.org/10.1038/nature10735.

33. Akhavan, A., Atanasiu, R., and Shrier, A. (2003). Identification of a COOH-terminal segment involved in maturation and stability of human ether-a-go-go-related gene potassium channels. J. Biol. Chem. *278*, 40105–40112. https://doi.org/10.1074/jbc.M307837200.

34. Jenke, M., Sánchez, A., Monje, F., Stühmer, W., Weseloh, R.M., and Pardo, L.A. (2003). C-terminal domains implicated in the functional surface expression of potassium channels. EMBO J. *22*, 395–403. https://doi.org/10.1093/emboj/cdg035.

35. Kupershmidt, S., Yang, T., Chanthaphaychith, S., Wang, Z., Towbin, J.A., and Roden, D.M. (2002). Defective human Ether-a-go-go-related gene trafficking linked to an endoplasmic reticulum retention signal in the C terminus. J. Biol. Chem. *277*, 27442–27448. https://doi.org/10.1074/jbc.M112375200.

36. Teng, S., Ma, L., Dong, Y., Lin, C., Ye, J., Bähring, R., Vardanyan, V., Yang, Y., Lin, Z., Pongs, O., and Hui, R. (2004). Clinical and electrophysiological characterization of a novel mutation R863X in HERG C-terminus associated with long QT syndrome. J. Mol. Med. *82*, 189–196. https://doi.org/10.1007/s00109-003-0504-1.

37. Roy, B., Friesen, W.J., Tomizawa, Y., Leszyk, J.D., Zhuo, J., Johnson, B., Dakka, J., Trotta, C.R., Xue, X., Mutyam, V., et al. (2016). Ataluren stimulates ribosomal selection of near-cognate tRNAs to promote nonsense suppression. Proc. Natl. Acad. Sci. USA *113*, 12508–12513. https://doi.org/10.1073/pnas.1605336113.

38. Kozek, K.A., Glazer, A.M., Ng, C.A., Blackwell, D., Egly, C.L., Vanags, L.R., Blair, M., Mitchell, D., Matreyek, K.A., Fowler, D.M., et al. (2020). High-throughput discovery of trafficking-deficient variants in the cardiac potassium channel K(V)11.1. Heart Rhythm *17*, 2180–2189. https://doi.org/10.1016/j.hrthm.2020.05.041.

39. Kanner, S.A., Jain, A., and Colecraft, H.M. (2018). Development of a High-Throughput Flow Cytometry Assay to Monitor Defective Trafficking and Rescue of Long QT2 Mutant hERG Channels. Front. Physiol. *9*, 397. https://doi.org/10.3389/fphys.2018.00397.

40. Dai, W.J., Zhu, L.Y., Yan, Z.Y., Xu, Y., Wang, Q.L., and Lu, X.J. (2016). CRISPR-Cas9 for *in vivo* Gene Therapy: Promise and Hurdles. Mol. Ther. Nucleic Acids *5*, e349. https://doi.org/10.1038/mtna.2016.58.

41. Peng, R., Lin, G., and Li, J. (2016). Potential pitfalls of CRISPR/Cas9-mediated genome editing. FEBS J. *283*, 1218–1231. https://doi.org/10.1111/febs.13586.

42. Wu, Z., Yang, H., and Colosi, P. (2010). Effect of genome size on AAV vector packaging. Mol. Ther. *18*, 80–86. https://doi.org/10.1038/mt.2009.255.

43. Wang, D., Tai, P.W.L., and Gao, G. (2019). Adeno-associated virus vector as a platform for gene therapy delivery. Nat. Rev. Drug Discov. *18*, 358–378. https://doi.org/10.1038/s41573-019-0012-9.

44. Mendell, J.R., Al-Zaidy, S.A., Rodino-Klapac, L.R., Goodspeed, K., Gray, S.J., Kay, C.N., Boye, S.L., Boye, S.E., George, L.A., Salabarria, S., et al. (2021). Current Clinical Applications of *In Vivo* Gene Therapy with AAVs. Mol. Ther. *29*, 464–488. https://doi.org/10.1016/j.ymthe.2020.12.007.

45. Schwartz, P.J., Crotti, L., and Insolia, R. (2012). Long-QT syndrome: from genetics to management. Circ. Arrhythm. Electrophysiol. *5*, 868–877. https://doi.org/10.1161/CIRCEP.111.962019.

OMTN, Volume *34*

Supplemental information

Transfer RNA-mediated restoration of potassium current and electrical correction in premature termination long-QT syndrome hERG mutants

Viggo G. Blomquist, Jacqueline Niu, Papiya Choudhury, Ahmad Al Saneh, Henry M. Colecraft, and Christopher A. Ahern

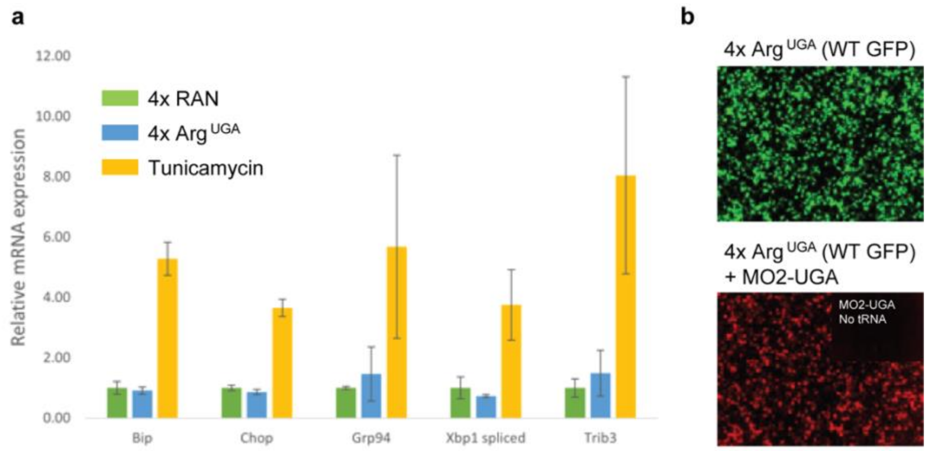

Figure S1. Expressed tRNA do not initiate UPR. a) HEK cells expressing the indicated construct were assayed by RT-PCR for UPR. 4X Arg^{UGA} contains four copies of an Arg^{UGA} suppressor, where 4X RAN has a randomized Arg^{UGA} tRNA cassette. Both plasmids contain a WT GFP to mark plasmid delivery. Tunicamycin is used as the positive control. b) upper, Expression of the 4X Arg^{UGA} GFP construct; lower, Arg^{UGA} tRNA activity as shown by rescue of an MO2-UGA plasmid.

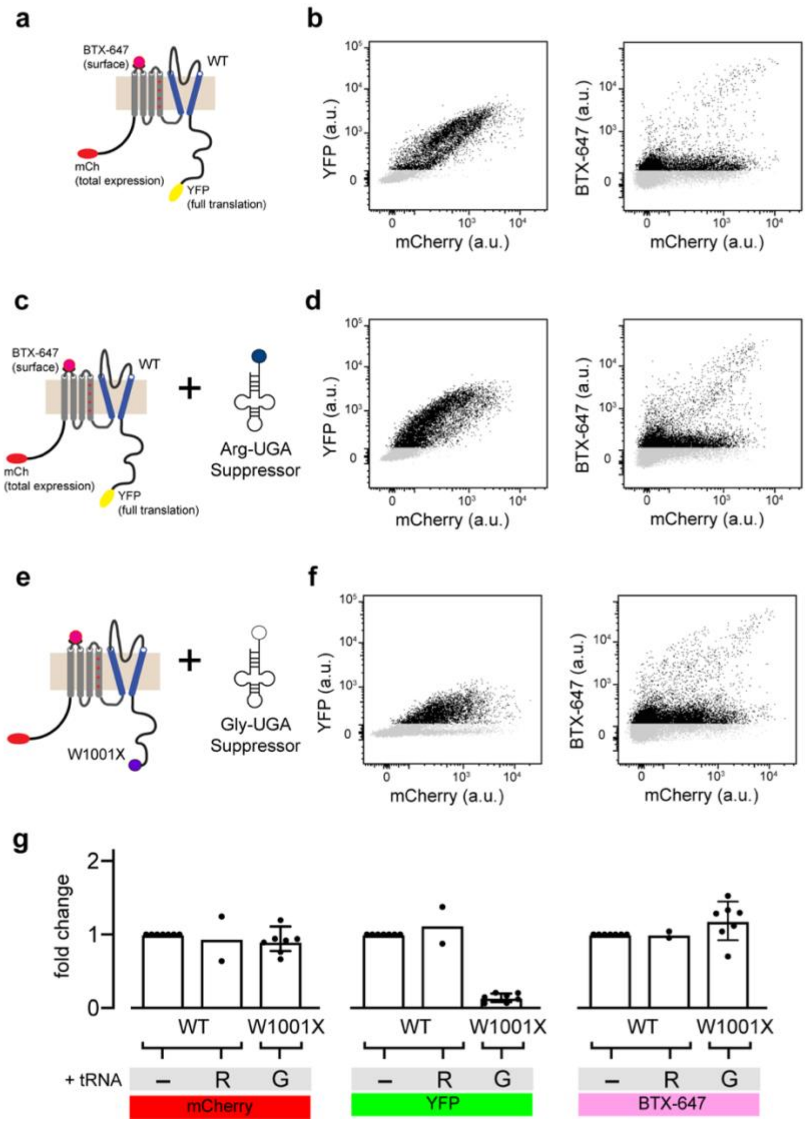

Figure S2. tRNA expression does not impact Wt hERG expression. a) Illustration of engineered WT hERG with mCherry fluorescence tag on the N-terminus, YFP on the C-terminus, and BTX-647 tagging the extracellular BTX binding site. b) Flow cytometric analysis of population distribution for mCherry, YFP, and BTX-647 fluorescence, where each cell is represented by a single point in a trial. YFP is plotted as a function of mCherry to show the relative proportion of channels fully translated (left). BTX-647 is plotted as a function of mCherry to show relative fraction of hERG trafficked to the plasma membrane (right). c) Cartoon depiction of hERG co-expressed Arg^{UGA} tRNA. d) Flow cytometric analysis of population distribution shows co-expression with tRNA does not affect mCherry (left) or channel trafficking (right). e) Cartoon depiction of hERG co-expressed Gly^{UGA} tRNA. f) Flow cytometric analysis of population distribution shows expression of tRNA restores full length hERG and YFP fluorescence (left) and has no effect on trafficking (right). g) Population summary of the geometric means of mCherry, YFP, and BTX-647 fluorescence for Wt hERG and W1001X co-expressed with tRNAs for each trial (where each trial includes >5000 cells). Error bars represent standard deviation (mean ± sd).

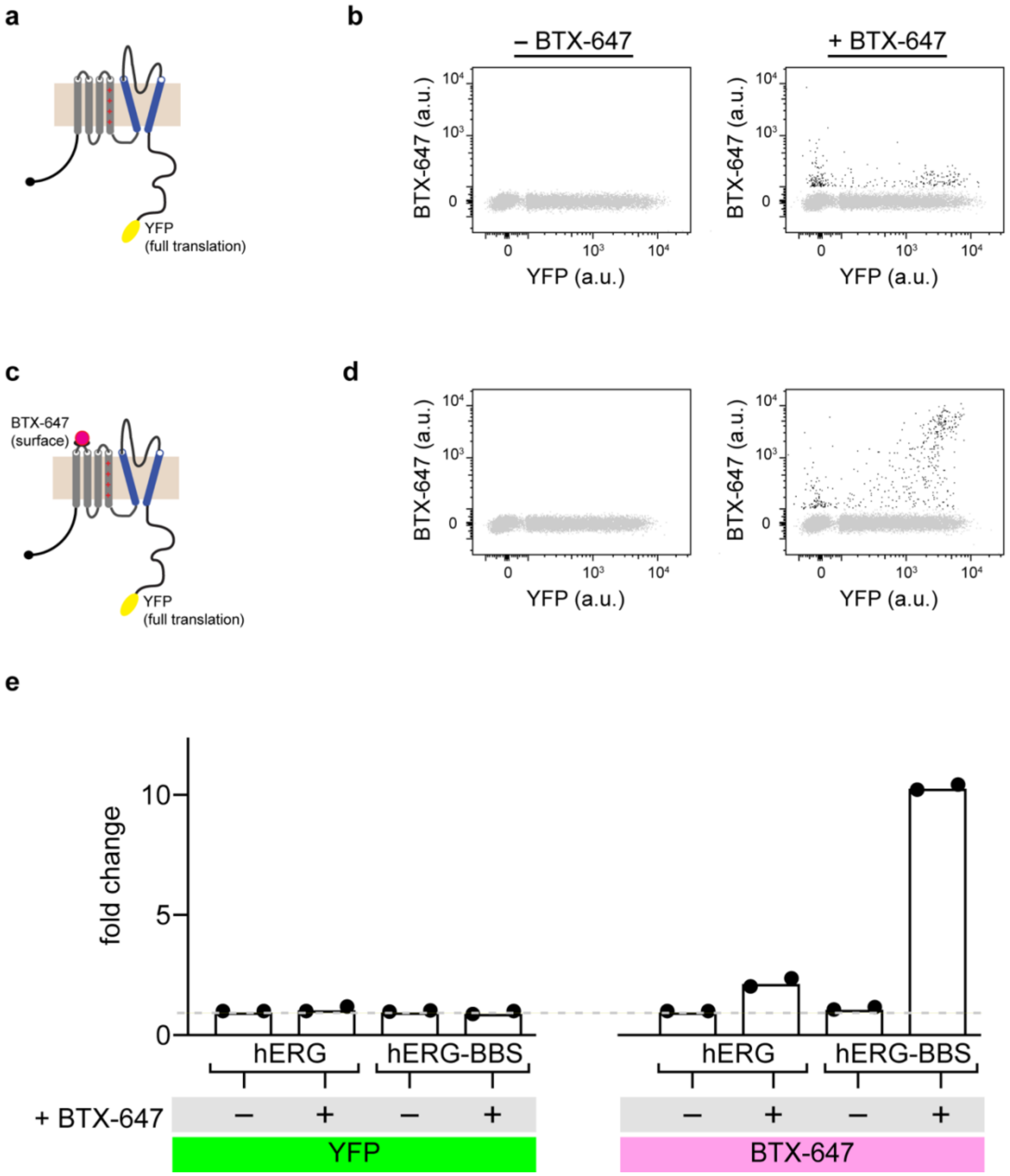

Figure S3. BTX-647 labeling is specific to BBS site in hERG. a) Illustration of engineered WT hERG with YFP fluorescence tag on the C-terminus. b) BTX-647 is plotted as a function of YFP to demonstrate any nonspecific signal without (left) or with (right) BTX-647 treatment. c) Illustration of engineered WT hERG with YFP fluorescence tag on the C-terminus and BTX-647 tagging the extracellular BTX binding site. d) BTX-647 is plotted as a function of YFP to demonstrate absence of membrane labeling without BTX-647 treatment (left) and clear labeling with BTX-647 treatment (right).

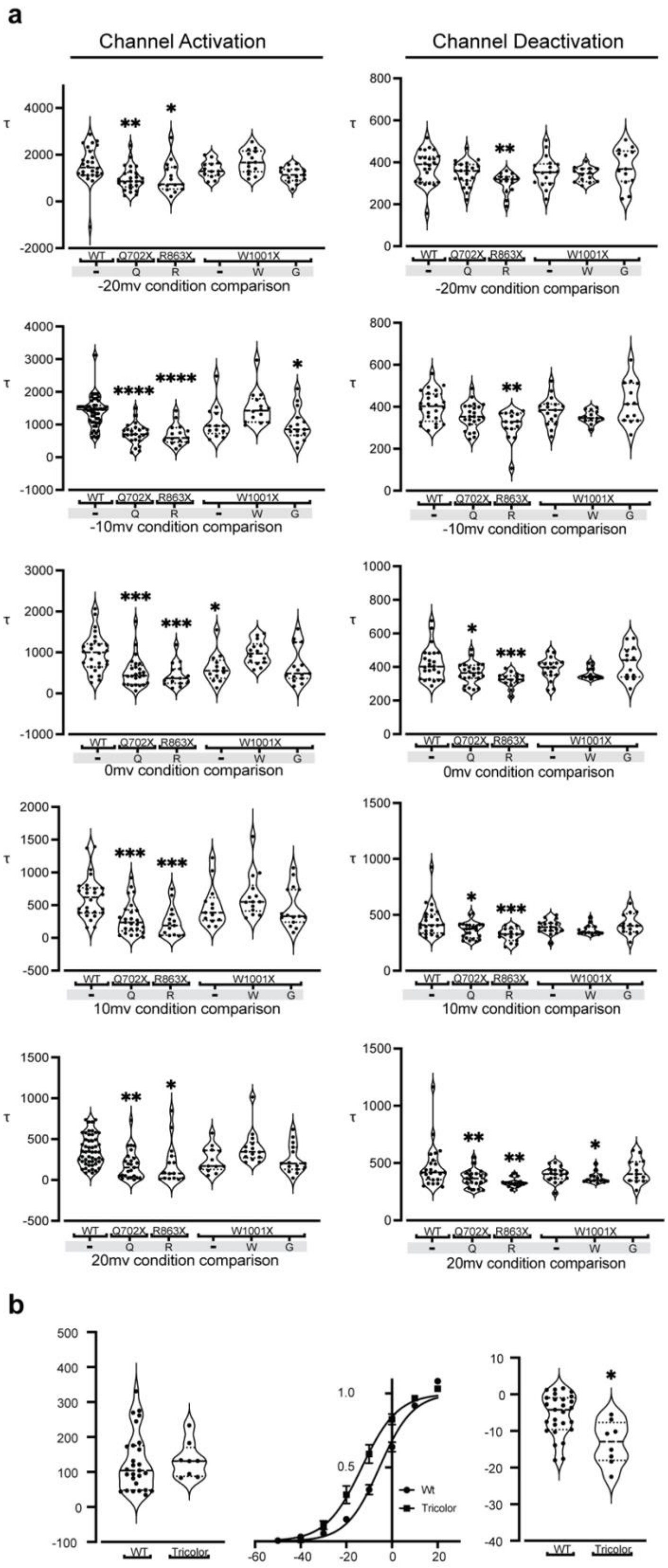

Figure S4. Further analysis of channel kinetics. a) hERG activation (left) and deactivation (right) at step voltages from -20mV to 20mV. A Dunnett's multiple comparison test was used to determine statistical significance (p>.05). b) WT and tricolor hERG current densities (left) current-voltage relationship (middle) and $V_{.05}$ values (right). An unpaired t-test was used to determine statistical significance (p>.05).

Table S1. GFP Rescue. Values are presented as mean ± SEM. The data in this table was used to generate Figure 1.

Condition (n)	GFP (normalized to Wt)
Wt GFP (3)	1.0 ± 0.0
GFP-TAG (3)	0.001 ± 0.0006
GFP-TAG + Gln tRNA (3)	0.3 ± 0.09
GFP-TGA (3)	0.002 ± 0.0005
GFP-TGA + Trp tRNA (3)	0.02 ± 0.005
GFP-TGA + Arg tRNA (3)	0.2 ± 0.05

Table S2. hERG expression, rescue, and trafficking. Values are presented as mean ± SEM. Statistical significance was set at $p<0.05$ and marked with asterisks. The data in this table was used to generate Figure 2 And Supplemental Figure S2.

Condition (n)	mCherry (normalized to WT)	YFP (normalized to WT)	BTX-647 (normalized to WT)
Wt hERG (11)	1.0 ± 0.0	1.0 ± 0.0	1.0 ± 0.0
Q702x (7)	2.0 ± 0.3*	0.0 ± 0.006	2.3 ± 0.2
Q702x + Gln tRNA (7)	1.0 ± 0.1	0.7 ± 0.07*	1.0 ± 0.08
R863X (7)	1.1 ± 0.1	0.0 ± 0.008	0.8 ± 0.06
R863X + Arg tRNA (7)	1.2 ± 0.1	0.6 ± 0.07*	1.1 ± 0.1
W1001X (9)	0.9 ± 0.07	0.009 ± 0.004	1.1 ± 0.1
W1001X + Trp tRNA (9)	0.9 ± 0.07	0.05 ± 0.008	1.2 ± 0.1
W1001X + Gly tRNA (9)	0.9 ± 0.05	0.1 ± 0.01	1.1 ± 0.08
Wt hERG + Arg tRNA (3)	0.9 ± 0.1	1.1 ± 0.1	1.1 ± 0.1

Table S3. Activation of rescued hERG. Values are presented as mean ± SEM. Statistical significance was set at $p<0.05$ and marked with asterisks.

Condition (n)	-20mV	-10mV	0mV	10mV	20mV
	τ activation (ms)	τ activation (ms)	τ activation (ms)	τ activation (ms)	τ activation (ms)
Wt hERG (26)	1549 ± 155.0	1408 ± 101.7	988.3 ± 91.4	620.4 ± 63.4	360.2 ± 36.6
Q702x + Gln tRNA (25)	976.0 ± 102.2*	721.0 ± 62.4*	521.6 ± 74.0*	300.2 ± 50.1*	187.8 ± 35.7*
R863X + Arg tRNA (15)	1032 ± 170.8*	697.5 ± 82.25*	446.5 ± 73.6*	237.3 ± 58.7*	194.0 ± 65.0*
W1001X (13)	1367 ± 100.4	1070 ± 145.6	619.8 ± 99.2*	490.6 ± 88.8	247.5 ± 42.9
W1001X + Trp tRNA (14)	1699 ± 132.9	1512 ± 139.2	989.4 ± 77.4	647.9 ± 88.7	399.0 ± 54.9
W1001X + Gly tRNA (15)	1134 ± 81.8	982.5 ± 128.7*	679.6 ± 115.6	461.6 ± 80.4	253.5 ± 45.2

Table S4. Deactivation of rescued hERG. Values are presented as mean ± SEM. Statistical significance was set at $p<0.05$ and marked with asterisks. The data in this table was used to generate Supplemental Figure S4.

Condition (n)	-20mV	-10mV	0mV	10mV	20mV
	τ deactivation (ms)	τ deactivation (ms)	τ deactivation (ms)	τ deactivation (ms)	τ deactivation (ms)
Wt hERG (24)	375.9 ± 16.3	400.9 ± 14.9	418.9 ± 20.3	442.9 ± 27.8	466.8 ± 37.9
Q702x + Gln tRNA (23)	347.6 ± 11.8	354.3 ± 12.9	362.2 ± 13.3	362.5 ± 14.9	367.0 ± 15.5
R863X + Arg tRNA (15)	302.7 ± 12.4	314.6 ± 18.3	324.2 ± 10.9	322.0 ± 15.6	330.3 ± 10.0
W1001X (17)	357.6 ± 17.3	381.3 ± 16.0	389.6 ± 16.2	390.3 ± 15.0	398.3 ± 16.2
W1001X + Trp tRNA (14)	342.0 ± 9.1	350.4 ± 7.9	363.3 ± 9.5	366.4 ± 12.2	371.9 ± 12.4
W1001X + Gly tRNA (13)	378.2 ± 25.1	419.0 ± 28.3	425.2 ± 26.8	417.3 ± 26.6	430.8 ± 29.3

Table S5. Action Potential Parameters I. Values are presented as mean ± SEM. The data in this table was used to generate Figure 4.

Condition	Resting Membrane Potential (mV)	Upstroke Velocity (V/s)	Action Potential Amplitude (mV)
blank	-69.0±4.0	88.8±72.1	112.1±27.9
WT	-61.9±2.2	85.1±40.2	103.5±18.4
R863X	-65.9±2.5	91.6±56.6	128.9±24.8
R863X+ArgtRNA	-56.5±1.8	72.9±38.4	98.1±14.0

Table S6. Action Potential Parameters: APD80 1 hz. Values are presented as mean ± SEM. Myocytes were paced at 1hz. One-way ANOVA ($P = 0.0736$) followed by Dunnett's multiple comparisons test was used to test statistical significance.

	Avg	SEM	n	Dunnett's Multiple Comparisons Test (p-values)
blank	437.11	72.41	6	0.5268
WT	339.71	37.97	7	n/a
R863X	622.50	134.5	3	0.0332
R863X+ArgtRNA	380.57	43.46	5	0.9399

Table S7. Tricolor hERG comparison to Wt hERG. Values are presented as mean ± SEM. Statistical significance was set at $p<0.05$ and marked with asterisks. The data in this table was used to generate Supplementary Figure S3.

Condition (n)	IV	Current Density
	$V_{1/2}$ (mV)	pA/pF
Wt hERG (27)	-5.5 ± 1.0	129.4 ± 16.1
Tricolor hERG (8)	-13.2 ± 2.0*	134.5 ± 18.2
